# NbCl_5_-Mg Reagent System in Regio- and Stereoselective Synthesis of (2*Z*)-Alkenylamines and (3*Z*)-Alkenylols from Substituted 2-Alkynylamines and 3-Alkynylols

**DOI:** 10.3390/molecules26123722

**Published:** 2021-06-18

**Authors:** Rita N. Kadikova, Azat M. Gabdullin, Oleg S. Mozgovoj, Ilfir R. Ramazanov, Usein M. Dzhemilev

**Affiliations:** Institute of Petrochemistry and Catalysis of Russian Academy of Sciences, 141 Prospekt Oktyabrya, 450075 Ufa, Russia; saogabdullinsao@gmail.com (A.M.G.); skill15@mail.ru (O.S.M.); ilfir.ramazanov@gmail.com (I.R.R.); ink@anrb.ru (U.M.D.)

**Keywords:** niobium(V) chloride, 2-alkenylamines, substituted 2-alkynylamines, magnesium metal, homopropargylic alcohols, methanesulfonyl chloride, chlorothiolation

## Abstract

The reduction of *N*,*N*-disubstituted 2-alkynylamines and substituted 3-alkynylols using the NbCl_5_–Mg reagent system affords the corresponding dideuterated (2*Z*)-alkenylamine and (3*Z*)-alkenylol derivatives in high yields in a regio- and stereoselective manner through the deuterolysis (or hydrolysis). The reaction of substituted propargylamines and homopropargylic alcohols with the in situ generated low-valent niobium complex (based on the reaction of NbCl_5_ with magnesium metal) is an efficient tool for the synthesis of allylamines and homoallylic alcohols bearing a 1,2-disubstituted double bond. It was found that the well-known approach for the reduction of alkynes based on the use of the TaCl_5_-Mg reagent system does not work for 2-alkynylamines and 3-alkynylols. Thus, this article reveals a difference in the behavior of two reagent systems—NbCl_5_-Mg and TaCl_5_-Mg, in relation to oxygen- and nitrogen-containing alkynes. A regio- and stereoselective method was developed for the synthesis of nitrogen-containing *E*-β-chlorovinyl sulfides based on the reaction of 2-alkynylamines with three equivalents of methanesulfonyl chloride in the presence of stoichiometric amounts of niobium(V) chloride and magnesium metal in toluene.

## 1. Introduction

The use of magnesium metal as a reducing agent for the generation of low-valent complexes of transition metals is well-described for numerous reactions giving titanocene and zirconocene alkyne complexes. Rosenthal and Burlakov [1,2] have performed studies on the synthesis of titanocene and zirconocene alkyne complexes based on the pioneering works done by Vol’pin and Shur [3,4]. The synthesized transition metal alkyne complexes are of value because these metallacyclopropenes are a source of reactive metallocenes serving as precursors for the synthesis of various organic compounds [1,4,5,6,7,8,9,10,11,12,13,14,15,16,17,18]. As regards the synthesis of niobium alkyne complexes, the chemistry of low-valent niobium complexes with unsaturated substrates is poorly known. Thus, methods for the synthesis of niobiacyclopropene complexes based on functionalized acetylenic compounds are lacking. Investigations of the chemistry of low-valent niobium often involve related tantalum complexes due to the similarity of their electronic and chemical properties. Niobium and tantalum alkyne complexes, as representatives of Group V metal complexes, were synthesized for the first time by Cotton and coworkers based on the reaction of sterically bulky alkynes with M_2_Cl_6_(THT)_3_ (M = Ta, Nb; THT is tetrahydrothiophene) giving dimeric MV complexes [19,20]. The synthesis of mononuclear niobium alkyne trihalides by the reactions of acetylenes with low-valent niobium complexes such as NbX_3_(DME) (X = Cl, Br) was reported by Roskamp [21]. The preparation of related trihaloniobium alkyne complexes by the reaction of alkynes with NbX_3_(DME), generated by the treatment of niobium(V) halides with tributyltin hydride, were described in the publications [21,22]. Five-membered niobacycles were prepared by the reaction of niobium alkyne complexes with allyl Grignard [23]. Niobium- and tantalum-containing complexes are of interest for organic synthesis as a simple and effective tool for obtaining amino alcohols [21], metallocyclic carbonyl derivatives [24], metallacyclic enamines [25] and vicinal diamines [26]. Low-valent niobium- and tantalum-containing complexes are known as effective catalysts for oligo- and polymerization of disubstituted acetylenes [20,27,28], chemoselective cycloaddition of alkynes to olefins [29,30,31] and nitriles [32,33]. One of the most commonly used approaches to the generation of low-valent transition metal compounds involves the reduction of high-valent metal salts with such metals as Zn, Mg, Al or Na/Hg amalgam. The synthesis of niobium alkyne complexes by the reduction of high-valent niobium salts with metals was performed using Al [34,35,36], Na/Hg amalgam [37,38,39,40,41] or Zn [42] as reducing agents. Based on the approaches to the preparation of niobium-alkyne complexes known in the literature, it can be assumed that the reduction reactions of alkynes using low-valence niobium complexes may be of interest for the selective preparation of olefinic compounds [43,44,45,46,47]. However, strangely enough, there are no published examples of the reduction of functionalized acetylenic compounds and non-functionalized alkynes using the reaction of alkynes with low-valent niobium complexes, generated by the treatment of niobium(V) chloride with magnesium metal. Meanwhile, the approach to the synthesis of tantalum alkyne complexes from tantalum(V) chloride and magnesium metal is well-developed [48]. To develop a new effective method for stereoselective reduction of alkynes using low-valent niobium complexes, we put forward the idea of the possibility of synthesizing intermediate niobiumcyclopropenes by reacting NbCl_5_ with metallic magnesium in the presence of 1,2-disubstituted acetylenes to obtain the target corresponding niobiumcyclopropenes. Hence, the goal of this work is to study the reaction of alkynes having different structures with the Mg–NbCl_5_ reagent system. Previously, we have developed a selective method for the EtMgBr–NbCl_5_-catalyzed reduction of substituted 2-alkynylamines with Et_2_Zn [49]. A limitation of this method is the impossibility of obtaining reduction products from acetylenic alcohols under organozinc synthesis conditions. Taking into account the stability of the amine function in the discovered niobium–magnesium-catalyzed transformation of tertiary 2-alkynylamines, it was of interest to study first and foremost the transformation of propargylamines using low-valent niobium, generated by the reaction of niobium(V) chloride with magnesium metal. Thus, this work is devoted to the study of the transformations of acetylene substrates under the action of the reagent system-Mg-NbCl_5_.

## 2. Results

**Scheme 1:**
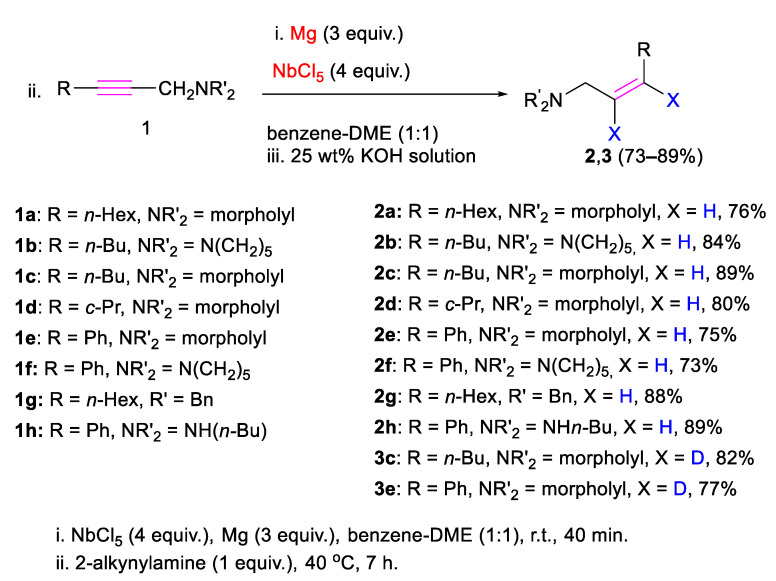
Reduction of substituted 2-alkynylamines to (*Z*)-2-alkenylamines with the Mg–NbCl_5_ reagent system. See the Supplementary Materials for details.

We found that the reaction of four equiv. NbC1_5_ with three equiv. of Mg in a 1:1 mixture of benzene and 1,2-dimethoxyethane (DME) at room temperature for 40 min followed by the addition of 2-alkynylamines **1** and by the heating at 40 °C for 7 h leads to the formation of reduction products **2a**–**h** and **3c**,**e** in high yields (73–89%) in a regio- and stereoselective manner through the hydrolysis (Scheme 1). Therefore, the presence of a nitrogen atom in the alkyne molecules does not hinder the formation of a niobium alkyne complex. We have now studied behavior of *N*-aryl-substituted 2-alkynylamines derivatives in the investigated reaction. We also found that the reduction of *N*,*N*-dibenzylnon-2-yn-1-amine **1g** under the action of the reagent system-Mg-NbCl_5_ affords reduction product **2g** in yields of 88% (Scheme 1). Thus, the presence of a benzyl substituent at the nitrogen atom in the structure of tertiary propargylamine **1g** does not prevent the reduction of the triple bond using the NbCl_5_-Mg reagent system. It follows from the performed experiment that the C-N-bond of the *N*-benzyl fragment is stable under the action of complexes of low-valent niobium. *N*-(Hept-2-yn-1-yl)-*N*-phenylaniline was not converted by the NbCl_5_-Mg reagent system. However, this NbCl_5_-Mg reagent system proved to be effective for the reduction of secondary 2-alkynyl amine that has a hydrogen atom at a nitrogen atom. In this way the reaction of *N*-(3-phenylprop-2-yn-1-yl)butan-1-amine **1h** with reagent system-Mg-NbCl_5_ affords reduction product **2h** in high yield (89%).

**Scheme 2:**
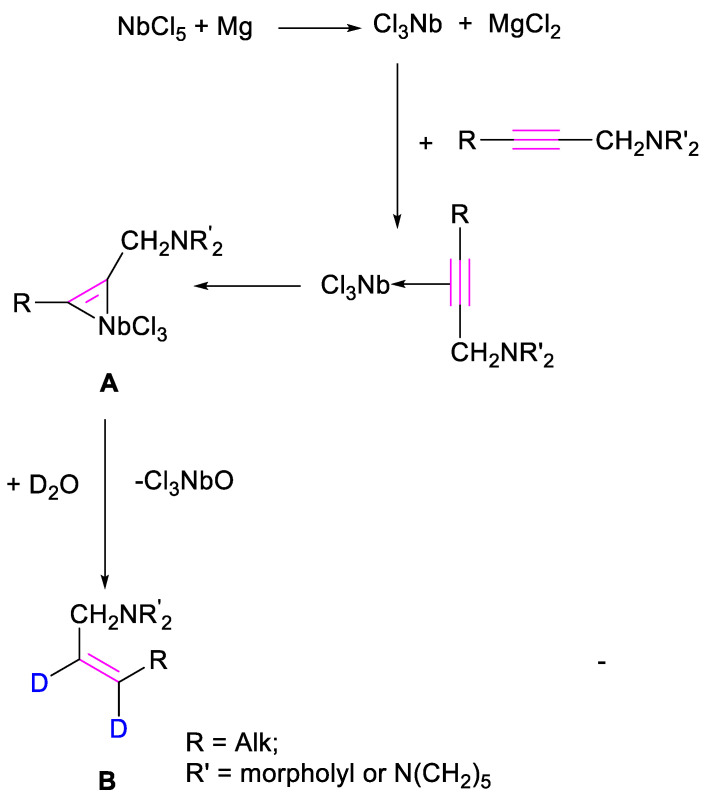
Putative mechanism of the reduction of 2-alkynylamines 1 with the Mg–NbCl_5_ reagent system.

The structural identification of the synthesized compounds was performed by 1D and 2D NMR spectroscopy. Based on the NOESY correlations, the *Z* configuration of the double bond was established for all hydrolysis products **2a**–**2h**. In particular, the NOESY spectra show nuclear Overhauser effects between the methylene group (δ 3.29–3.31) at the sp^2^-hybridized carbon atom of the double bond and phenyl protons (δ 7.20–7.38), which is indicative of the formation of diastereomer **2f** with the *Z* configuration of the double bond. The absence of signals of carbon atoms at δ 123.39–137.80 in the ^13^C NMR spectra of deuterolysis products **3c** and **3e** attests to the presence of two deuterium atoms at the double bond. The presence of two vicinal deuterium atoms at the double bond in the dideuterated allylamines may be indicative of the formation of the niobiacyclopropene intermediate **A** (Scheme 2). The reduction of niobium(V) chloride to a niobium(III) compound with mercury-activated magnesium metal was described in the study [50]. The formation of a niobium(III) complex was also observed in the reaction of Nb(ŋ-C_5_Me_5_)Cl_4_ with magnesium metal [51]. On the other hand, the TaCl_5_-catalyzed carbomagnesiation of alkenes with n-alkyl Grignard reagents is initiated by the generation of the tantalum(III) chloride alkene complex [52,53,54]. Hence, we suggested that the reaction of niobium(V) chloride with magnesium metal also leads to the generation of a low-valent niobium(III) complex. The further coordination of the latter by a propargylamine molecule affords the organoniobium intermediate **A** (Scheme 2). The deuterolysis of the complex **A** gives dideuterated (2*Z*)-alkenylamine **B**. The reaction of 4-(non-2-yn-1-yl)morpholine **1a** with 20 mol.% of NbCl_5_ and three equivalents of magnesium metal in a 1:1 mixture of benzene and 1,2-dimethoxyethane (DME) at room temperature, followed by heating to 40 °C for 7 h resulted in the formation of reduction product in minor amounts. Apparently niobiumcyclopropenes can be considered reaction intermediates. The hydrolysis of the latter gives allylamines.

Initially, we performed experiments in a two-component solvent system consisting of benzene and 1,2-dimethoxyethane. It is well known that the generation of Ta and Nb alkyne complexes via the reactions of TaCl_5_ with Mg and of NbCl_5_ with Zn in a mixture of benzene and DME occurs with high selectivity and in high yields [48]. We used this two-component solvent system taking into account good solubility of tantalum and niobium halides and the corresponding organometallic intermediates in a mixture of benzene and 1,2-dimethoxyethane. Besides, 1,2-dimethoxyethane is commonly employed as the solvent in the generation of niobium alkyne complexes due to the stabilizing effect of DME molecules on niobium complexes through the coordination of the oxygen atoms of 1,2-dimethoxyethane to niobium. This imparts high thermal stability to Nb and Ta alkyne complexes and makes the transformation of Group V metal alkyne complexes into different classes of organic compounds easy and simple, as opposed to related Ti and Zr complexes. Titanium(II) and zirconium(II) alkyne complexes are the best-known low-valent early transition metal complexes with alkynes. However, many alkyne complexes involving TiII and ZrII are thermally unstable and cannot be used in further reactions [33]. It should also be noted that NbCl_3_(DME) is a thermally stable and commercially available complex. Such low-valent niobium species have been used both as reagents and as catalysts in organic transformations. We studied the effect of the solvent nature on the possibility of the reduction of 2-alkynylamines under the organoniobium synthesis conditions. The possibility of the reduction of the triple bound under the conditions of organoniobium synthesis in different solvents was investigated in relation to the reaction of 4-(non-2-yn-1-yl)morpholine **1a** with 4 equiv. of NbCl_5_ and 3 equiv. of Mg (Table 1).

**Scheme 3:**
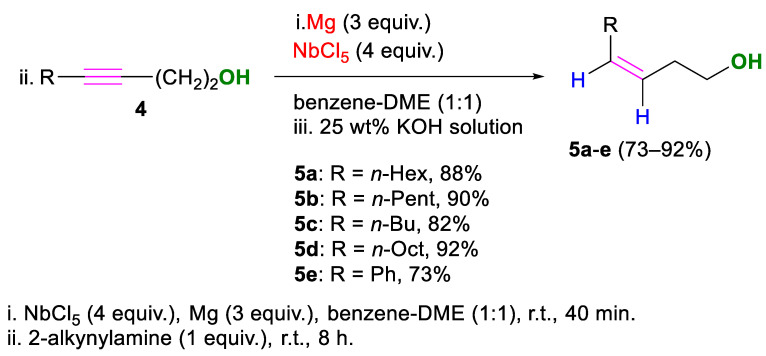
Reduction of substituted 3-alkynols with the NbCl_5_–Mg reagent system.

The reduction of 4-(non-2-yn-1-yl)morpholine was found to occur efficiently not only in the DME–benzene solvent system but also in the diethyl ether–benzene system giving allylamine **2a** in 81% yield (entry 2). It is worth noting that the commonly employed methods for the reduction of non-functionalized alkynes with TaCl_5_–Zn, TaCl_5_–Mg and NbCl_5_–Zn reagent systems are generally accomplished in DME–benzene or DME–toluene solvent systems [48,55]. Also, 2-Alkynylamine is efficiently reduced to **2a** in a dichloromethane–benzene mixture (Entry 3). Therefore, the reduction of 2-alkynylamines with the NbCl_5_–Mg reagent system does not require the presence of an ethereal solvent. However, the use a polar chlorine-containing solvent is accompanied by an insignificant decrease in the yield of the reduction product due to side reactions giving an unidentified mixture of high-molecular-weight compounds, which are apparently polymerization products of 2-alkynylamine involving low-valent niobium complexes (according to the gas chromatographic analysis, the yield of **2a** was 72%; entry 3). Meanwhile, in the reaction using pure toluene, benzene or DME as the solvent, the conversion of the starting propargylamine was less than 5%, and **2b** was obtained in trace amounts (entries 4, 5 and 6). The observed low conversion of propargylamine in the reaction under consideration performed in aromatic solvents is apparently attributed to poor solubility of chlorine-containing niobium complexes in toluene and benzene. It was found that 2-alkynylamines are not reduced with the TaCl_5_–Mg reagent system. The reaction of 4-(non-2-yn-1-yl)morpholine 1a with four equiv. of TaCl_5_ and three equiv. of Mg in a 1:1 benzene–DME solvent mixture by the heating to 40 °C for 7 h gives the reduction product in trace amounts. Therefore, the presence of the oxygen-containing morpholyl substituent in 2-alkynylamine does not hinder the selective reduction of the triple bond (Scheme 1). In the next step of our study concerning the reduction of functionalized acetylenic compounds under the organoniobium synthesis conditions, we investigated the reaction of substituted acetylenic alcohols with the NbCl_5_–Mg reagent system. We failed to reduce the triple bond in such propargylic alcohols as non-2-yn-1-ol and 3-phenylprop-2-yn-1-ol under the organoniobium synthesis conditions. The starting acetylenic substrates and their transformation products were not detected in the reaction mixture by gas chromatography. These experimental data suggest that the transformation of propargylic alcohols is accompanied by the oligo- or polymerization. Apparently, in the case of propargylic alcohols, the formation of high-molecular-weight compounds is attributed to the polymerization of niobium-containing allene, which is generated via the β-elimination of the alkoxide group of the niobiacyclopropene intermediate. Previously, we have found that the Zr-catalyzed cycloalumination of substituted propargylic alcohols was also accompanied by the polymerization as the side reaction caused by the β-elimination of the alkoxide group [56]. However, the reaction of substituted homopropargylic alcohols **4** with four equiv. of NbCl_5_ and three equiv. of Mg in a 1:1 mixture of benzene and DME performed at room temperature for 8 h affords reduction products **5a**–**e** in high yields (73–92%) in a regio- and stereoselective manner through the hydrolysis (Scheme 3). It should be emphasized that the reduction of homopropargylic alcohols is inhibited when using TaCl_5_ instead of NbCl_5_. The conversion of dec-3-yn-1-ol **5a** in the reduction reaction under the conditions presented in Scheme 3 is less than 5%. As can be seen in Scheme 3, the transformation of homopropargylic alcohols **4** into unsaturated alcohols **5** does not require warming of the reaction mixture to 40 °C, as opposed to propargylamines **1** (Scheme 1). Therefore, 3-alkynylols are more reactive than 2-alkynylamines in the reduction of alkynes with the NbCl_5_–Mg reagent system. Apparently, the more distal position of the hydroxyl substituent not only promotes the selective reduction of alkinols under the action of NbCl_5_-Mg, preventing β-elemination of the hydroxyl group, but also an increase in reactivity in comparison with 2- alkynyl amines. However, this system is unsuitable for the reduction of dialkyl-substituted acetylenes. The reaction of 5-decyne (or diphenylacetylene) with NbCl_5_(4 equiv.) and Mg (3 equiv.) in a mixture of benzene and DME (1:1) by the heating to 40 °C for 7 h gives the reduction products in trace amounts.

**Scheme 4:**
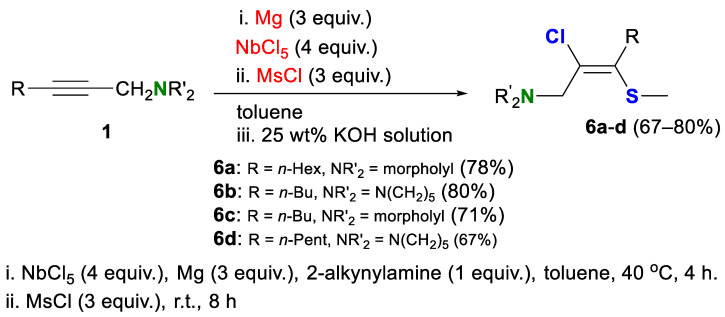
Chlorothiolation of alkynes with the NbCl_5_–Mg reagent system.

In order to further study transformations of acetylenic compounds under the organoniobium synthesis conditions, we made attempts to develop efficient reagents for transformations of niobiacyclopropene intermediates. Previously, we have demonstrated that sulfonic acid derivatives, such as sulfonyl halides, sulfonic acid silyl esters, alkylthiosulfonates, organic disulfides and diselenides are efficient electrophilic reagents for the functionalization of organoaluminum compounds, such as 1-alkenylalanes, which can be used to prepare the corresponding alkenyl halides [57], alkenylsilanes [58], alkenyl sulphides [59,60] and alkenyl selenides [61] in high yields under mild conditions. We expected that sulfonyl halides would also be reactive toward niobiacyclopropene intermediate generated in this reaction. However, all our attempts to perform the reaction of niobiacyclopropene, prepared by the reduction of **1a** (Scheme 1), with methanesulfonyl chloride, failed. Thus, the reaction of the organoniobium intermediate (prepared by the reduction of 4-(non-2-yn-1-yl)morpholine **1a** with four equiv. of NbCl_5_ and three equiv. of Mg in a mixture of benzene and DME) with three equiv. of methanesulfonyl chloride (MsCl) at room temperature followed by alkaline hydrolysis afforded, within 7 h, exclusively reduction product **2a** in 82% yield. Meanwhile, the reaction in a 1:1 mixture of diethyl ether and benzene also did not lead to the desired functionalization of the organoniobium intermediate with MsCl. As described above, 2-alkynylamines can be reduced with the NbCl_5_–Mg reagent system in a mixture of dichloromethane and benzene (Table 1, entry 3). The reaction with three equivalents of methanesulfonyl chloride (MsCl) in a 1:1 mixture of dichloromethane and benzene, performed at room temperature for 8 h, gives (*E*)-4-(2-chloro-3-(methylthio)non-2-en-1-yl)morpholine **6a** as the chlorothiolation product in 18% isolated yield. In this case, the formation of the chlorothiolation product with the *E* configuration of the double bond and the absence of cross-coupling products with MsCl in two-component solvents, such as DME–benzene and Et_2_O–benzene mixtures, cast doubt on the involvement of the putative niobiacyclopropene intermediate in the chlorothiolation. We found that the reaction of 4-(non-2-yn-1-yl)morpholine **1a** with four equiv. of NbCl_5_ and three equiv. of Mg in toluene followed by the addition of three equiv. of MsCl at room temperature is accompanied by the formation of (*E*)-4-(2-chloro-3-(methylthio)non-2-en-1-yl)morpholine **6a** in 78% yield within 8 h through the alkaline hydrolysis. The conversion of the starting propargylamine **1a** was 88% (Scheme 4).

An increase in the reaction time and the use of a higher concentration (6 equiv.) of methanesulfonyl chloride did not lead to the complete conversion of the starting alkyne. However, as described above, the reduction of 4-(non-2-yn-1-yl)morpholine **1a** with the NbCl_5_–Mg reagent system is inhibited in toluene (entry 4, Table 1). The observed inertness of 2-alkynylamine in the reduction reaction under consideration in toluene is an additional argument in favor of the suggestion that the niobiacyclopropene intermediate is not involved in the reaction with MsCl and that the observed transformation proceeds through another nontrivial mechanism. It was found that stoichiometric amounts of NbCl_5_ and Mg are required for the transformation of the starting propargylamine using MsCl. Meanwhile, the transformation of the starting alkyne requires the addition of MsCl (three equiv.) to a mixture of 2-alkynylamine, NbCl_5_, and magnesium metal in toluene pre-heated at 40 °C for 4 h. Apparently, the increase in the temperature to 40 °C is necessary for the generation of the low-valent metal complex reactive in the chlorothiolation of propargylamines. For example, it is known that the formation of NbCl_4_ in the reaction of niobium(V) halides with aluminum metal under reduced pressure requires a temperature of 250 °C [62]. Here, we report the experimental data on the chlorothiolation of alkynes only for propargylamines. Besides, we found that the reactions of 5-decyne and 4-octyne with methanesulfonyl chloride in toluene afford p-tolylthiolation products, which is indicative of the direct involvement of the solvent molecules in the reaction. Hence, the reaction of sulfonyl chlorides having different structures with other heteroatom-containing alkynes and non-functionalized alkynes requires a special study.

**Scheme 5:**
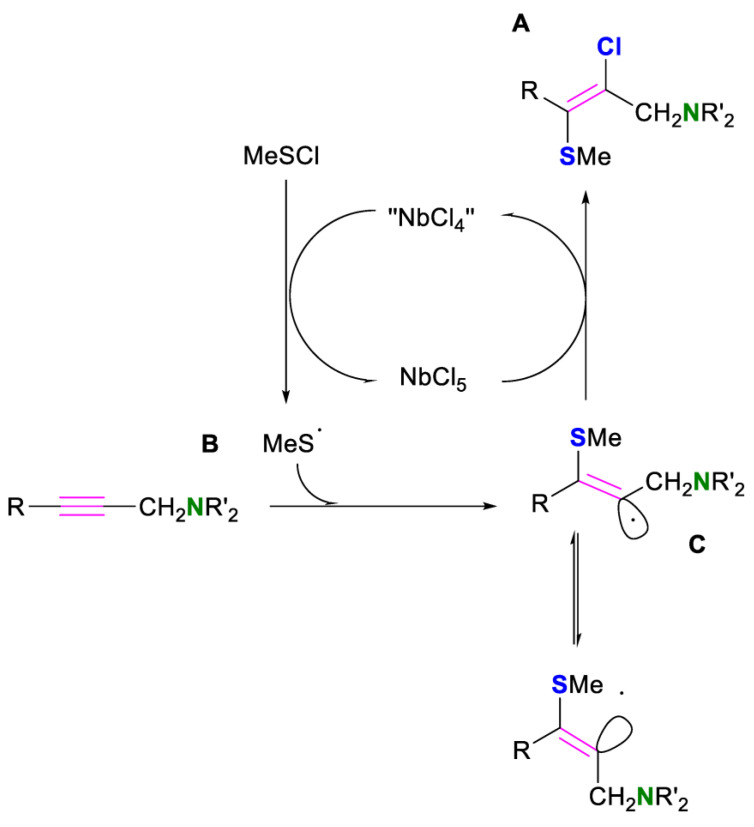
Chlorothiolation of alkynes with the NbCl_5_–Mg reagent system.

Scheme 5 shows the reaction pathway of the chlorothiolation of 2-alkynylamines. Apparently, the sulfonic group is reduced to the sulfide moiety with the NbCl_5_–Mg reagent system (Scheme 5, Equation (1)). According to the literature data [63,64,65], the addition of the sulfonyl radical to the triple bond is the key step in the Cu-catalyzed chlorothiolation of acetylenes and the FeCl_2_-catalyzed chlorosulfonation of 1-alkynyl chlorides [66]. Hence, we suggested that the observed transformation can be initiated by the complex “NbCl_4_” **A**, which is generated in the reduction of NbCl_5_ with magnesium metal in toluene (Scheme 5, Equation (2)). The paramagnetic complex “NbCl_4_” A can, in turn, initiate the S–Cl bond homolysis of sulfenyl chloride giving the sulfenyl radical **B** and NbCl_5_. Monomeric low-valent niobium coordination compounds, such as NbCl_4_L_2_, are known to be paramagnetic [67]. On the other hand, as can be seen in Table 1, the reaction of 2-alkynylamine **1a** with the NbCl_5_–Mg reagent system in toluene afforded reduction product **2a** in minor amounts (chromatography mass-spectrometry data), which is indicative of the inhibition of the pathway toward the putative niobiacyclopropene intermediate. The addition of the sulfenyl radical **B** to 2-alkynylamine is accompanied by the formation of the alkyl radical **C**. The latter is transformed into β-haloalkenyl sulfide accompanied by the regeneration of “NbCl_4_”. A similar trans-addition of sulfonyl chlorides to the triple bond was described in the literature [63,68,69].

(1) $${\mathrm{MeSO}}_{2}\mathrm{Cl}\overset{{\mathrm{NbCl}}_{5}\;-\;\mathrm{Mg}}{\to }\mathrm{MeSCl}$$

(2) $${\mathrm{NbCl}}_{5}+\mathrm{Mg}\to {“\mathrm{NbCl}}_{4}”\;+{\mathrm{MgCl}}_{2}$$

The new method for the synthesis of β-haloalkenyl sulfides, along with the electrophilic chlorothiolation of terminal alkynes by sulfenyl chloride [70], bromothiolation with sulfenyl bromine [71], and the Pd- and Fe-catalyzed regio- and stereoselective addition of sulfenyl chlorides to terminal acetylenes [72,73], is of high value because the halogen atom can be easily transformed into different alkenyl sulfide derivatives.

## 3. Materials and Methods

### 3.1. General Information

**Figure 1:**
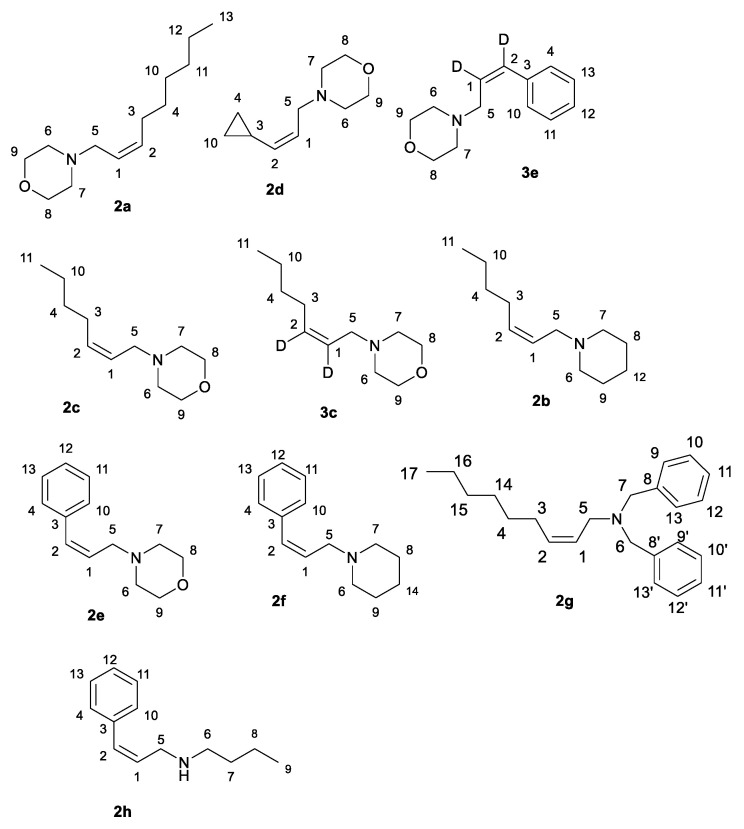
The numbering of atoms in the ^13^C- and ^1^H-NMR spectra of the compounds **2a**–**h**, **3c**,**e**.

**Figure 2:**
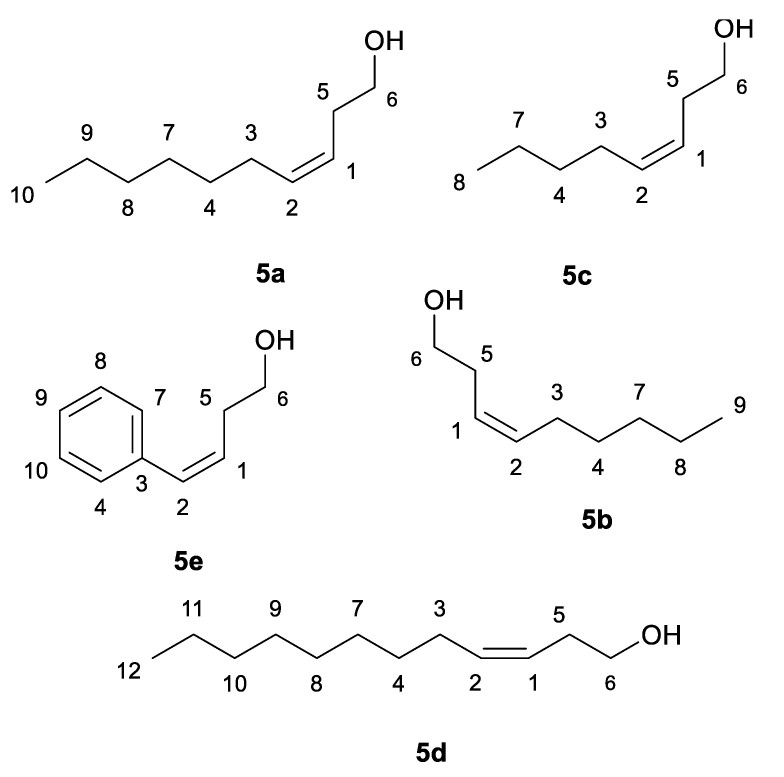
The numbering of atoms in the ^13^C- and ^1^H-NMR spectra of the compounds **5a**–**e**.

**Figure 3:**
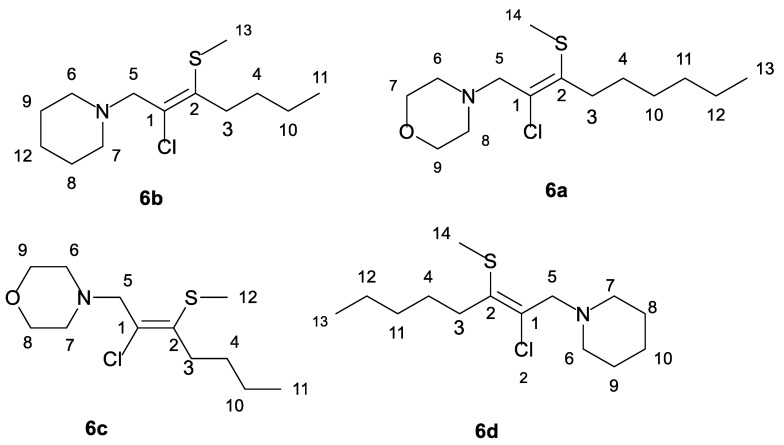
The numbering of atoms in the ^13^C- and ^1^H-NMR spectra of the compounds **6a**–**d**.

The reagents were obtained from Sigma-Aldrich or Acros. Dichloromethane were distilled over P_2_O_5_. Diethyl ether, benzene, toluene and 1,2-dimethoxyethane were dried over sodium. 2-Alkynylamines **1a**–**h** were prepared by aminomethylation of terminal alkynes with aqueous formaldehyde and secondary amines under CuI catalysis [74]. 3-Alkynylols **4** were prepared by the reaction of alkynylmagnesium reagents with ethylene oxide [75]. IR spectra were recorded on Bruker VE Vertex 70v spectrometer as liquid films or in Nujol and are reported in wavenumbers (cm^−1^). Nuclear magnetic resonance spectroscopy was performed on a Brucker Avance 500. The ^1^H NMR spectra were recorded at 500 MHz and ^13^C-{^1^H} NMR spectra at 125 MHz in CDCl_3_. The chemical shifts are reported in ppm relative to tetramethylsilane (TMS) as the internal standard. The numbering of atoms in the ^13^C-{^1^H} and ^1^H NMR spectra of the compounds **2a**–**h**, **3c**,**e**, **5a**–**e**, **6a**–**d** is shown in Figure 1, Figure 2 and Figure 3. Elemental analysis was performed using a Carlo-Erba CHN 1106 elemental analyzer. Mass spectra were obtained on a Finnigan 4021 instrument. The yields were calculated from the isolated amount of allylamines obtained from starting 2-alkynylamines.

### 3.2. Preparation of (Z)-2-Alkenylamines ***2a–h***, ***3c***,***e*** via Reduction of Substituted 2-Alkynylamines via Mg-NbCl_5_

(*Z*)-4-(non-2-en-1-yl)morpholine; Typical Procedure

In a 50-mL reaction flask was placed NbC1_5_ (2160 mg, 8 mmol) under an argon atmosphere. To the salt benzene was added at room temperature (12 mL) and DME (12 mL), successively. Magnesium powder (144 mg, 6 mmol) was added to the stirring pale yellow solution of NbC1_5_ and the resulting mixture was stirred at room temperature for 40 min. To the mixture was added at room temperature of a 4-(non-2-yn-1-yl)morpholine (418 mg, 2.0 mmol) and the whole mixture was stirred at 40 °C 7 h. After 7 h at 40 °C, the reaction mixture was diluted with Et_2_O (20 mL), and 25 wt% KOH solution (15 mL) was added dropwise while the reaction flask was cooled in an ice bath. The aqueous layer was extracted with diethyl ether (3 × 20 mL). The combined organic layers were washed with brine (20 mL), dried over anhydrous MgSO_4_. The reaction mixture was filtered through a filter paper and concentrated in vacuo to give crude product as a yellow oil. The residue was distilled through a micro column at 2.4 mmHg to give **2a** (321 mg, 76%) as a colorless oil. b.p. 126–127 °C (2.4 mmHg). ^1^H NMR (500 MHz, CDCl_3_): δ = 0.86 (t, *J* = 6 Hz, 3H, C(13)H_3_), 1.22–1.28 (m, 6H, C(10–12)H_2_), 1.29–1.32 (m, 2H, C(4)H_2_), 2.04 (q, *J* = 7 Hz, 2H, C(3)H_2_), 2.43 (s, 4H, C(6, 7)H_2_), 2.98 (d, *J* = 7 Hz, 2H, C(5)H_2_), 3.69 (t, *J* = 3 Hz 4H, C(8, 9)H_2_), 5.39–5.44 (m, 1H, C(1)H), 5.52–5.57 (m, 1H, C(2)H). ^13^C NMR (125MHz, CDCl_3_): δ = 14.04 (C(13)), 22.59 (C(12)), 27.49 (C(3)), 28.90 (C(10)), 29.46 (C(4)), 31.68 (C(11)), 55.47 (C(5)), 53.61 (C(6, 7)), 66.99 (C(8, 9)), 125.32 (C(1)), 133.75 (C(2)). MS (EI): *m*/*z*, % = 211 (3) [M^+^], 126 (5), 87 (100), 86 (40), 57 (30), 40 (15). Anal. calcd for C_13_H_25_NO, (%): C, 73.88; H, 11.92; N, 6.63; Found, %: C, 74.83; H, 11.88; N, 6.57.

(*Z*)-1-(hept-2-en-1-yl)piperidine (**2b**)

Using the procedure described above 358 mg of 1-(hept-2-yn-1-yl)piperidine (2 mmol) gave crude product that was distilled through a micro column at 3.4 mmHg to afford **2b** (304 mg, 84%) as a colorless oil. b.p. 107–110 °C (3.4 mmHg). ^1^H NMR (500 MHz, CDCl_3_): δ = 0.90 (t, *J* = 7 Hz, 3H, C(11)H_3_), 1.32–1.35 (m, 4H, C(4, 10)H_2_), 1.44 (s, 2H, C(12)H_2_), 1.59–1.63 (m, 4H, C(8, 9)H_2_), 2.06 (q, *J* = 6 Hz, 2H, C(3)H_2_), 2.42 (s, 4H, C(6, 7)H_2_), 3.01 (d, *J* = 6 Hz, 2H, C(5)H_2_), 5.46–5.51 (m, 1H, C(1)H), 5.52–5.57 (m, 1H, C(2)H). ^13^C NMR (125 MHz, CDCl_3_): δ = 13.96 (C(11)), 22.31 (C(10)), 24.26 (C(12)), 25.81 (C(8, 9)), 27.19 (C(3)), 31.72 (C(4)), 54.34 (C(6, 7)), 55.68 (C(5)), 125.91 ((C(1)), 133.15 (C(2)). MS (EI): *m*/*z*, % = 181 (7) [M^+^], 138 (4), 124 (10), 98 (29), 84 (100), 55 (30), 41 (15). Anal. calcd for C_12_H_23_N, (%): C, 79.49; H, 12.79; N, 7.72; Found, %: C, 79.45; H, 12.83; N, 7.52.

(*Z*)-4-(hept-2-en-1-yl)morpholine (**2c**)

Using the procedure described above 362 mg of 4-(hept-2-yn-1-yl)morpholine (2 mmol) gave crude product that was distilled through a micro column at 5 mmHg to afford **2c** (326 mg, 89%) as a colorless oil. b.p. 109–111 °C (5 mmHg). ^1^H NMR (500 MHz, CDCl_3_): δ = 0.82 (t, *J* = 6 Hz, 3H, C(11)H_3_), 1.25–1.29 (m, 4H, C(4, 10)H_2_), 1.99 (q, *J* = 7 Hz, 2H, C(5)H_2_), 3.64 (s, 4H, C(8, 9)H_2_), 5.35–5.39 (m, 1H, C(1)H), 5.47–5.52 (m, 1H, C(2)H). ^13^C NMR (125 MHz, CDCl_3_): δ = 13.85 (C(11)), 22.21 (C(10)), 27.12 (C(3)), 31.61 (C(4)), 55.36 (C(5)), 53.49 (C(6, 7)), 66.83 (C(8, 9)), 125.17 ((C(1)), 133.68 (C(2)). MS (EI): *m*/*z*, % = 183 (10) [M^+^], 140 (4), 110 (28), 87 (100), 57 (70), 41 (21). Anal. calcd for C_11_H_21_NO, (%): C, 72.08; H, 11.55; N, 7.64; Found, %: C, 72.22; H, 11.56; N, 7.37.

(*Z*)-4-(3-cyclopropylallyl)morpholine (**2d**)

Using the procedure described above 330 mg of 4-(3-cyclopropylprop-2-yn-1-yl)morpholine (2 mmol) gave crude product that was distilled through a micro column at 4 mmHg to afford **2d** (267 mg, 80%) as a colorless oil. b.p. 91–93 °C (4 mmHg). ^1^H NMR (500 MHz, CDCl_3_): δ = 0.29–0.32 (m, 2H (A), C(4, 10)H_2_), 0.69–0.74 (m, 2H (B), C(4, 10)H_2_), 1.55–1.57 (m, 1H, C(3)H), 2.46 (s, 4H, C(6, 7)H_2_), 3.09 (d, *J* = 7 Hz, 2H, C(5)H_2_), 3.69 (t, *J* = 4 Hz, 4H, C(8, 9)H_2_), 5.32–5.37 (m, 1H, C(1)H), 4.89 (t, *J* = 10 Hz, 1H, C(2)H). ^13^C NMR (125 MHz, CDCl_3_): δ = 6.97 (C(4, 10)), 9.78 (C(3)), 53.62 (C(6, 7)), 55.86 (C(5)), 67.01 (C(8, 9)), 123.39 (C(1)), 137.80 (C(2)). MS (EI): *m*/*z*, % = 167 (10) [M^+^], 138 (33), 87 (70), 79 (87), 56 (69), 40 (100). Anal. calcd for C_10_H_17_NO, (%): C, 71.81; H, 10.25; N, 8.37; Found, %: C, 71.98; H, 10.35; N, 8.35.

(*Z*)-4-(3-phenylallyl)morpholine (**2e**)

Using the procedure described above 330 mg of 402 mg of 4-(3-phenylprop-2-yn-1-yl)morpholine (2 mmol) gave crude product that was distilled through a micro column at 1 mmHg to afford **2e** (305 mg, 75%) as a colorless oil. b.p. 130–132 °C (1 mmHg). ^1^H NMR (500 MHz, CDCl_3_): δ = 2.49 (s, 4H, C(6, 7)H_2_), 3.29–3.31(dd, J = 2 Hz, *J* = 6 Hz, 2H, C(5)H_2_), 3.75 (t, *J* = 4 Hz, 4H, C(8, 9)H_2_), 5.78–5.83 (m, 1H, C(1)H), 6.63 (d, *J* = 11 Hz, 1H, C(2)H), 7.26–7.29 (m, 3H, C(4, 10, 12)H), 7.37 (t, *J* = 8 Hz, 2H, C(11, 13)H). ^13^C NMR (125 MHz, CDCl_3_): δ = 53.71 (C(6, 7)), 59.05 (C(5)), 67.03 (C(8, 9)), 126.97 (C(12)), 128.17 (C(11, 13)), 128.89 (C(4, 10)), 129.00 (C(1)), 131.69 (C(2)), 137.00 (C(3)). MS (EI): *m*/*z*, % = 203 (20) [M^+^], 172 (4), 144 (12), 117 (72), 112 (100), 91 (33), 56 (32). Anal. calcd for C_13_H_17_NO, (%): C, 76.81; H, 8.43; N, 6.89; O, 7.87; Found, %: C, 76.90; H, 8.37; N, 7.01.

(*Z*)-1-(3-phenylallyl)piperidine (**2f**)

Using the procedure described above 398 mg of 1-(3-phenylprop-2-yn-1-yl)piperidine (2 mmol) gave crude product that was distilled through a micro column at 1 mmHg to afford **2f** (293 mg, 73%) as a colorless oil. b.p. 122–124 °C (1 mmHg). ^1^H NMR (500 MHz, CDCl_3_): δ = 1.45 (C(14)), 1.59–1.64 (m, 4H, C(8, 9)H_2_), 2.42 (s, 4H, C(6, 7)H_2_), 3.28 (d, *J* = 6 Hz, 2H, C(5)H_2_), 5.82–5.87 (m, 1H, C(1)H), 6.57 (d, *J* = 12 Hz, 1H, C(2)H), 7.24–7.28 (m, 3H, C(4, 10, 12)H), 7.33–7.37 (m, 2H, C(11, 13)H). ^13^C NMR (125 MHz, CDCl_3_): δ = 24.29 (C(14)), 26.01 (C(8, 9)), 54.70 (C(6, 7)), 57.12 (C(5)), 126.75 (C(12)), 128.09 (C(11, 13)), 128.91 (C(4, 10)), 130.31 (C(1)), 130.75 (C(2)), 137.29 (C(3)). MS (EI): *m*/*z*, % = 201 (12) [M^+^], 200 (15), 117 (44), 115 (38), 110 (100), 98 (30), 84 (12). Anal. calcd for C_14_H_19_N, (%): C, 83.53; H, 9.51; N, 6.96; Found, %: C, 83.61; H, 9.47; N, 7.12.

(*Z*)-4-(hept-2-en-1-yl-2,3-d_2_)morpholine (**3c**)

Using the procedure described above 362 mg of 4-(hept-2-yn-1-yl)morpholine (2 mmol) and D_2_O gave crude product that was distilled through a micro column at 2.4 mmHg to afford **3c** (303 mg, 82%) as a colorless oil. b.p. 118–120 °C (2.4 mmHg). IR (liquid film): 2958, 2925, 2870, 2855, 2811, 1742, 1742, 1654, 1618, 1519, 1508, 1456, 1399, 1118, 1070, 1034, 1008, 943, 866, 801 cm^−1^. ^1^H NMR (500 MHz, CDCl_3_): δ = 0.91 (t, *J* = 6 Hz, 3H, C(11)H_3_), 1.34–1.38 (m, 4H, C(4, 10)H_2_), 2.08 (t, *J* = 6 Hz, 2H, C(3)H_2_), 2.47 (s, 4H, C(6, 7)H_2_), 3.03 (s, 2H, C(5)H_2_), 3.74 (s, 4H, C(8, 9)H_2_). ^13^C NMR (125 MHz, CDCl_3_): δ = 13.96 (C(11)), 22.32 (C(10)), 27.09 (C(3)), 31.69 (C(4)), 53.61 (C(6, 7)), 55.36 (C(5)), 67.01 (C(8, 9)). MS (EI): *m*/*z*, % = 185 (7) [M^+^], 156 (1), 128 (6), 112 (19), 87 (100), 57 (70), 57 (70), 42 (13). Anal. calcd for C_11_H_19_D_2_NO, (%): C, 71.30; N, 7.56; Found, %: C, 71.46; N, 7.42.

(*Z*)-4-(3-phenylallyl-2,3-d_2_)morpholine (**3e**)

Using the procedure described above 402 mg of 4-(3-phenylprop-2-yn-1-yl)morpholine (2 mmol) and D_2_O gave crude product that was distilled through a micro column at 1 mmHg to afford **3e** (316 mg, 77%) as a colorless oil. b.p. 130–132 °C (1 mmHg). IR (liquid film): 3021, 2959, 2926, 2855, 2807, 2759, 1519, 1493, 1454, 1316, 1294, 1216, 1117, 1007, 778, 700, 669, 598, 472 cm^−1^. ^1^H NMR (500 MHz, CDCl_3_): δ = 2.49 (s, 4H, C(6, 7)H_2_), 3.29 (s, 2H, C(5)H_2_), 3.74 (t, *J* = 4 Hz, 4H, C(8, 9)H_2_), 7.26–7.29 (m, 3H, C(4, 10, 12)H), 7.37 (t, *J* = 8 Hz, 2H, C(11, 13)H). ^13^C NMR (125 MHz, CDCl_3_): δ = 53.72 (C(6, 7)), 56.51 (C(5)), 67.02 (C(8, 9)), 126.97 (C(12)), 128.17 (C(11, 13)), 128.89 (C(4, 10)), 136.93 (C(3)). MS (EI): *m*/*z*, % = 205 (21) [M^+^], 204 (14), 146 (9), 119 (73), 113 (100), 86 (19), 56 (32). Anal. calcd for C_13_H_15_D_2_NO, (%): C, 76.06; N, 6.82. Found, %: C, 76.13; N, 6.95.

(*Z*)-*N*,*N*-dibenzylnon-2-en-1-amine (**2g**)

Using the procedure described above 638 mg of *N*,*N*-dibenzylnon-2-yn-1-amine (2 mmol) and H_2_O gave crude product that was distilled through a micro column at 1 mmHg to afford **2g** (567 mg, 88%) as a colorless oil. b.p. 193–195 °C (1 mmHg). ^1^H NMR (500 MHz, CDCl_3_): δ = 0.97 (t, *J* = 4 Hz, 3H, C(17)H_3_), 1.33–1.43 (m, 8H, C(4, 5, 14, 15)H_2_), 2.04–2.08 (m, 2H, C(3)H_2_), 3.14–3.16 (m, 2H, C(5)H_2_), 3.64 (s, 4H, C(6, 7)H_2_), 5.62–5.65 (m, 2H, C(1, 2)H), 7.28–7.32 (m, 2H, C(11, 11′)H), 7.37–7.39 (m, 4H, C(10, 12, 10′, 12′)H), 7.44–7.48 (m, 4H, C(9, 13, 9′, 13′)H). ^13^C NMR (125 MHz, CDCl3): δ = 14.18 (C(17)), 22.71 (C(16)), 27.65 (C(3)), 29.04 (C(4)), 29.66 (C(14)), 31.80 (C(15)), 50.13 (C(5)), 58.03 (2C(6, 7)), 126.82 (3C (1, 11, 11′)), 128.19 (4C (10, 12, 10′, 12′), 128.87 (4C (9, 13, 9′, 13′), 133.22 (C(2)), 139.88 (2C (8, 8′)). MS (EI): *m*/*z*, % = 322 (<1) [M^+^], 232 (3), 210 (59), 181 (4), 91 (100), 65 (4). Anal. calcd for C_23_H_31_N, (%): C, 85.92; H, 9.72; N, 4.36. Found, %: C, 86.86; H, 9.68; N, 4.10.

(*Z*)-N-(3-phenylallyl)butan-1-amine (**2h**)

Using the procedure described above 374 mg of *N*-(3-phenylprop-2-yn-1-yl)butan-1-amine (2 mmol) and H_2_O gave crude product that was distilled through a micro column at 3 mmHg to afford **2h** (336 mg, 89%) as a colorless oil. b.p. 130–132 °C (3 mmHg). ^1^H NMR (500 MHz, CDCl_3_): δ = 0.93 (t, *J* = 7 Hz, 3H, C(9)H_3_), 1.32–1.39 (m, 2H, C(8)H_2_), 1.46–1.52 (m, 2H, C(7)H_2_), 2.65 (t, *J* = 7 Hz, 2H, C(6)H_2_), 3.57 (d, *J* = 6 Hz, 2H, C(5)H_2_), 5.78–5.83 (m, 1H, C(1)H), 6.55 (d, *J* = 12 Hz, 1H, C(2)H), 7.25–7.28 (m, 3H, C(4, 10, 12)H), 7.34–7.37 (t, *J* = 7 Hz, 2H, C(11, 13)H). ^13^C NMR (125 MHz, CDCl_3_): δ = 13.99 (C(9)), 20.48 (C(8)), 32.14 (C(7)), 47.71(C(5)), 49.34 (C(6)), 126.89 (C(12)), 128.17 (2C(11, 13)), 128.78 (2C(4, 10)), 130.41 (C(2)), 131.15 (C(1), 137.12 (C(3)). MS (EI): *m*/*z*, % = 189 (7) [M^+^], 146 (10), 117 (100), 91 (14), 84 (10). Anal. calcd for C_13_H_19_N, (%): C, 82.48; H, 10.12; N, 7.40. Found, %: C, 82.35; H, 10.01; N, 7.33.

### 3.3. Preparation of (3Z)-Alkenylols ***5a–e*** via Reduction of Substituted Alkynylols via Mg-NbCl_5_

(*Z*)-dec-3-en-1-ol; Typical Procedure

In a 50-mL reaction flask was placed NbC1_5_ (2160 mg, 8 mmol) under an argon atmosphere. To the salt was added at room temperature benzene (12 mL) and DME (12 mL), successively. Magnesium powder (144 mg, 6 mmol) was added to the stirring pale yellow solution of NbC1_5_ and the resulting mixture was stirred at room temperature for 40 min. To the mixture was added at room temperature of a dec-3-yn-1-ol (616 mg, 3.0 mmol) and the whole mixture was stirred at room temperature 8 h. After 8 h at room temperature, the reaction mixture was diluted with Et_2_O (20 mL), and 25 wt% KOH solution (15 mL) was added dropwise while the reaction flask was cooled in an ice bath. The aqueous layer was extracted with diethyl ether (3 × 20 mL). The combined organic layers were washed with brine (20 mL), dried over anhydrous MgSO_4_. The reaction mixture was filtered through a filter paper and concentrated in vacuo to give crude product as a yellow oil. The residue was distilled through a micro column at 4 mmHg to give **5a** (275 mg, 88%) as a colorless oil. b.p. 99–101 °C (4 mmHg). ^1^H NMR (500 MHz, CDCl_3_): δ = 0.90 (t, *J* = 6 Hz, 3H, C(10)H_3_), 1.27–1.29 (m, 8H, C(4, 7, 8, 9)H_2_), 2.04–2.06 (m, 2H, C(3)H_2_), 2.35 (q, *J* = 7 Hz, 2H, C(5)H_2_), 3.66 (t, *J* = 6 Hz, 2H, C(6)H_2_), 5.36–5.41 (m, 1H, C(1)H), 5.56–5.61 (m, 1H, C(2)H). ^13^C NMR (125 MHz, CDCl_3_): δ = 14.09 (C(10)), 22.63 (C(9)), 27.38 (C(3)), 29.67 (C(4)), 29.70 (C(7)), 30.90 (C(5)), 31.75 (C(8)), 62.37 (C(6)), 124.93 (C(1)), 133.58 (C(2)). MS (EI): *m*/*z*, % = 170 (3) [M^+^], 111 (11), 95 (25), 83 (47), 69 (74), 55 (100), 41 (46). Anal. calcd for C_10_H_20_O, (%): C, 76.86; H, 12.90; Found, %: C, 77.04; H, 12.83.

(*Z*)-non-3-en-1-ol (**5b**)

Using the procedure described above 280 mg of non-3-yn-1-ol (2 mmol) gave crude product that was distilled through a micro column at 10 mmHg to afford **5b** (256 mg, 90%) as a colorless oil. b.p. 93–95 °C (10 mmHg). The spectral properties (^1^H NMR, ^13^C NMR, MS) were in good agreement with those that were reported in the literature [76].

(*Z*)-oct-3-en-1-ol (**5c**)

Using the procedure described above 252 mg of oct-3-yn-1-ol (2 mmol) gave crude product that was distilled through a micro column at 14 mmHg to afford **5c** (210 mg, 82%) as a colorless oil. b.p. 88–90 °C(14 mmHg). ^1^H NMR (500 MHz, CDCl_3_): δ = 0.93 (t, *J* = 7 Hz, 3H, C(8)H_3_), 1.39–1.44 (m, 2H, C(7)H_2_), 1.47–1.53 (m, 2H, C(4)H_2_), 2.19 (t, *J* = 7 Hz, 2H, C(3)H_2_), 2.44–2.47 (m, 2H, C(5)H_2_), 3.69 (br. s, 2H, C(6)H_2_), 5.36–5.40 (m, 2H, C(1)H), 5.53–5.61 (m, 1H, C(2)H). ^13^C NMR (125 MHz, CDCl_3_): δ = 13.61 (C(8)), 18.92 (C(3)), 21.96 (C(7)), 23.19 (C(5)), 31.07 (C(4)), 61.39 (C(6)), 124.93 (C(1)), 133.56 (C(2)). Anal. calcd for C_8_H_16_O, (%): C, 74.94; H, 12.58; Found, %: C, 75.12; H, 12.67.

(*Z*)-dodec-3-en-1-ol (**5d**)

Using the procedure described above 362 mg of dodec-3-yn-1-ol (2 mmol) gave crude product that was distilled through a micro column at 2 mmHg to afford **5d** (339 mg, 92%) as a colorless oil. b.p. 115–117 °C (2 mmHg). The spectral properties (^1^H NMR, ^13^C NMR, MS) were in good agreement with those that were reported in the literature [77].

(*Z*)-4-phenylbut-3-en-1-ol (**5e**)

Using the procedure described above 292 mg of 4-phenylbut-3-yn-1-ol (2 mmol) gave crude product that was distilled through a micro column at 1.8 mmHg to afford **5e** (216 mg, 73%) as a colorless oil. b.p. 117–121 °C (1.8 mmHg). ^1^H NMR (500 MHz, CDCl_3_): δ = 2.64 (q, *J* = 7 Hz, 2H, C(5)H_2_), 3.74 (t, *J* = 6 Hz, 2H, C(6)H_2_), 5.71–5.76 (m, 1H, C(1)H), 6.61 (d, *J* = 12 Hz, 1H, C(2)H), 7.23–7.29 (m, 1H, C(9)H), 7.33–7.39 (m, 4H, C(4, 7, 8, 10)H). ^13^C NMR (125 MHz, CDCl_3_): δ = 32.02 (C(5)), 62.37 (C(6)), 126.83 (C(8)), 128.46 (C(1)), 131.36 (C(2)), 128.26 (C(8, 10)), 128.78 (C(4, 7)), 137.32 (C(3)). MS (EI): *m*/*z*, % = 148 (40) [M^+^], 117 (100), 115 (95), 104 (89), 91 (66), 77 (9), 65 (15). Anal. calcd for C_10_H_12_O (%): C, 81.04; H, 8.16. Found, %: C, 80.73; H, 8.19.

### 3.4. Preparation of (3Z)-Alkenylols ***6a–d*** via Chlorothiolation of Alkynes with the NbCl_5_–Mg Reagent System

(*E*)-4-(2-chloro-3-(methylthio)non-2-en-1-yl)morpholine; Typical Procedure

In a 50-mL reaction flask was placed NbC1_5_ (2160 mg, 8 mmol) under an argon atmosphere. To the salt was added at room temperature benzene (12 mL) and DME (12 mL) successively. Magnesium powder (144 mg, 6 mmol) and a 4-(non-2-yn-1-yl)morpholine (418 mg, 2.0 mmol) were added to the stirring pale yellow solution of NbC1_5_ the resulting mixture was stirred at 40 °C for 4 h. After 4 h at 40 °C to the mixture was added at room temperature of a methanesulfonyl chloride (690 mg, 6.0 mmol) and the whole mixture was stirred at room temperature 8 h. After 8 h at room temperature, the reaction mixture was diluted with Et_2_O (20 mL), and 25 wt% KOH solution (15 mL) was added dropwise while the reaction flask was cooled in an ice bath. The aqueous layer was extracted with diethyl ether (3 × 20 mL). The combined organic layers were washed with brine (20 mL), dried over anhydrous MgSO_4_. The reaction mixture was filtered through a filter paper and concentrated in vacuo to give crude product as a yellow oil. The residue was distilled through a micro column at 1.6 mmHg to give **6a** (456 mg, 78%) as a colorless oil. b.p. 97–100 °C (1.6 mmHg). ^1^H NMR (500 MHz, CDCl_3_): δ = 0.91 (t, *J =* 7 Hz, 3H, C(13)H_3_), 1.33 (s, 6H, C(12, 11, 10)H2), 1.56–1.60 (m, 2H, C(4)), 2.36 (s, 3H, C(14)H_3_), 2.52 (t, *J =* 7 Hz, 4H, C(6, 7)H_2_), 2.74 (t, *J =* 7 Hz, 2H, C(3)H_2_), 3.42 (s, 2H, C(5)H_2_), 3.73 (t, *J =* 4 Hz, C(8, 9)). ^13^C NMR (125 MHz, CDCl_3_): δ = 14.05 (C(13)), 16.35 (C(14)), 22.57 (C(12)), 27.49 (C(4)), 28.50 (C(10)), 31.58 (C(11)), 37.23(C(3)), 53.19 (C(6, 7)), 59.09 (C(5)), 67.08 (C(8, 9)), 127.42 (C(2)), 136.95(C(1))MS (EI): *m*/*z*, % = 292 (<1) [M^+^], 291 (2), 189 (<1), 100 (100), 86 (7). Anal. calcd for C_14_H_26_ClNOS, (%): C, 57.41; H, 8.98; N, 4.80. Found, %: C, 57.74; H, 8.83; N, 4.89.

(*E*)-1-(2-chloro-3-(methylthio)hept-2-en-1-yl)piperidine (**6b**)

Using the procedure described above 358 mg of 1-(hept-2-yn-1-yl)piperidine (2 mmol) gave crude product that was distilled through a micro column at 2 mmHg to afford **6b** (419 mg, 80%) as a colorless oil. b.p. 97–99 °C (2 mmHg). ^1^H NMR (500 MHz, CDCl_3_): δ = 0.96 (t, *J =* 7 Hz, 3H, C(11)H_3_), 1.37 (q, *J =* 7 Hz, 2H, C(10)H_2_), 1.44–1.47 (m, 2H, C(12)), 1.55–1.61 (m, 6H, C(4, 8, 9)H_2_), 2.36 (s, 3H, C(13)H_3_), 2.43 (s, 4H, C(6, 7)H_2_), 2.74 (t, *J =* 7 Hz, 2H, C(3)H_2_), 3.36 (s, 2H, C(5)H_2_). ^13^C NMR (125 MHz, CDCl_3_): δ = 13.95 (C(11)), 16.35 (C(13)), 22.02 (C(10)), 24.45 (C(12)), 26.06 (C(8, 9)), 29.72 (C(4)), 36.98 (C(3)), 54.13 (C(6, 7)), 59.63 (C(5)), 128.52 (C(2)), 135.89 (C(1)). MS (EI): *m*/*z*, % = 262 (1) [M^+^], 261 (4), 178 (1), 98 (100), 84 (12). Anal. calcd for C_13_H_24_ClNS, (%): C, 59.63; H, 9.24; N, 5.35. Found, %: C, 59.78; H, 9.41; N, 5.59.

(*E*)-4-(2-chloro-3-(methylthio)hept-2-en-1-yl)morpholine (**6c**)

Using the procedure described above 362 mg of 4-(hept-2-yn-1-yl)morpholine (2 mmol) gave crude product that was distilled through a micro column at 1.9 mmHg to afford **6c** (375 mg, 71%) as a colorless oil. b.p. 128–130 °C (1.9 mmHg). ^1^H NMR (500 MHz, CDCl_3_): δ = 0.96 (t, *J =* 7 Hz, 3H, C(11)H_3_), 1.34–1.39 (m, 2H, C(10)H_2_), 1.55–1.61 (m, 2H, C(4)H_2_), 2.36 (s, 3H, C(12)H_3_), 2.52 (s, 4H, C(6, 7)H_2_), 2.75 (t, *J =* 7 Hz, 2H, C(3)H_2_), 3.42 (s, 2H, C(5)H_2_), 3.74 (s, 4H, C(8, 9)H_2_). ^13^C NMR (125 MHz, CDCl_3_): δ = 13.92 (C(11)), 16.35 (C(12)), 22.00 (C(10)), 29.69 (C(4)), 36.99 (C(3)), 53.17 (C(6, 7)), 59.07 (C(5)), 67.06 (C(8, 9)), 127.41 (C(2)), 136.89 (C(1)). MS (EI): *m*/*z*, % = 264 (13) [M^+^], 263 (65), 230 (18), 228 (19), 176 (10), 101 (98), 100 (100), 86 (47), 56 (29). Anal. calcd for C_12_H_22_ClNOS, (%): C, 54.63; H, 8.41; N, 5.31. Found, %: C, 54.78; H, 8.21; N, 5.29.

## 4. Conclusions

To conclude, we have demonstrated for the first time that the NbCl_5_–Mg reagent system is an efficient tool for the regio- and stereoselective reduction of substituted 2-alkynylamines and 3-alkynylols to the corresponding (2*Z*)-alkenylamines and (3*Z*)-alkenylols. However, substituted homopropargylic alcohols and 2-alkynylamines do not undergo a transformation when treated with stoichiometric amounts of tantalum(V) chloride and magnesium metal. Thus, we have demonstrated for the first time the efficiency of the NbCl_5_-Mg reagent system for the reduction of 2-alkynylamines and 3-alkynylols. It was found that a similar method known in the literature for the reduction of alkynes using TaCl_5_-Mg does not work for such functionally substituted alkynes as 2-alkynylamines and 3-alkynylols. We also reported the first results of the study of the reaction between 2-alkynylamines and methanesulfonyl chloride in the presence of stoichiometric amounts of NbCl_5_ and Mg. A regio- and stereoselective method was developed for the synthesis of nitrogen-containing *E*-β-chlorovinyl sulfides based on the reaction of 2-alkynylamines with methanesulfonyl chloride in the presence of stoichiometric amounts of niobium(V) chloride and magnesium metal in toluene. In connection with the discovered selective reduction of functionally substituted alkynes using the NbCl_5_-Mg reagent system, we plan to study the reaction of phosphorus- and sulfur-containing alkynes with NbCl_5_ in the presence of metallic magnesium. One of the interesting findings of this article is the reaction of chlorothiolation of 2-alkynyl amines with methanesulfonyl chloride in the presence of stoichiometric amounts of NbCl_5_-Mg. The observed transformation indicates the generation of paramagnetic niobium complexes. This finding can serve as a basis for the development of a new method for the synthesis of multisubstituted olefins using the reaction of reductive coupling of carbonyl compounds under the action of paramagnetic niobium complexes. The observed tolerance of nitrogen- and oxygen-containing substituents under the conditions of organoniobium synthesis can serve as a basis for the development of effective methods for the synthesis of functionalized polysubstituted olefins.

## Figures and Tables

**Table 1:** Effect of the solvent nature on the reduction of 2-alkynylamines with the NbCl_5_–Mg reagent system ^a^.

Entry	Solvent	Conversion of 2-Alkynylamine (%)	Yield of 2a (%)
1	DME ^b^: Benzene ^b^	>99	89
2	Et_2_O ^b^: Benzene ^b^	>80	81
3	CH_2_Cl_2_ ^b^: Benzene ^b^	>99	72
4	Toluene	<5	4 (GC)
5	Benzene	<5	3 (GC)
6	DME	<5	<1 (GC)

^a^ reaction conditions: i. NbCl_5_ (4 equiv.), Mg (3 equiv.), solvent, r.t., 40 min. ii. 2-alkynylamine (1 equiv.), 40 °C, 7 h. iii. 25 wt% KOH solution. ^b^ solvent 1: solvent 2 (1:1).

## Data Availability

The data used to support the findings of this study are included within the article and supplementary materials.

## Supplementary Materials

The following are available online, analytical data and NMR spectra for all compounds.

## Sample Availability

Samples of the compounds are available from the authors.
