# Supplementary material for: NbCl5-Mg Reagent System in Regio- and Stereoselective Synthesis of (2Z)-Alkenylamines and (3Z)-Alkenylols from Substituted 2-Alkynylamines and 3-Alkynylols
Source: Molecules. 2021 Jun 18;26(12):3722. doi: 10.3390/molecules26123722 (PMC8234592; doi:10.3390/molecules26123722)

*Rita N. Kadikova, Azat M. Gabdullin, Oleg S. Mozgovoj, Ilfir R. Ramazanov, Usein M. Dzhemilev

$NbCl_5$-Mg reagent system in regio- and stereoselective synthesis of (2*Z*)-alkenylamines and (3*Z*)-alkenylols from substituted 2-alkynylamines and 3-alkynylols

Institute of Petrochemistry and Catalysis of Russian Academy of Sciences, 141 Prospekt Oktyabrya, Ufa 450075, Russian Federation

Tel./fax: +7-347-284-2750

E-mail address: kadikritan@gmail.com

Supporting information

General information

The reagents were obtained from Sigma-Aldrich or Acros. Dichloromethane were distilled over P_2O_5. Diethyl ether, benzene, toluene and 1,2-dimethoxyethane were dried over sodium. 2-Alkynylamines **1a-h** were prepared by aminomethylation of terminal alkynes with aqueous formaldehyde and secondary amines under CuI catalysis [1]. 3-Alkynylols **4** were prepared by the reaction of alkynylmagnesium reagents with ethylene oxide [2]. IR spectra were recorded on Bruker VE Vertex 70v spectrometer as liquid films or in Nujol and are reported in wavenumbers (cm^{-1}). Nuclear magnetic resonance spectroscopy was performed on a Brucker Avance 500. The ^1H NMR spectra were recorded at 500 MHz and ^{13}C-{^1H} NMR spectra at 125 MHz in $CDCl_3$. The chemical shifts are reported in ppm relative to tetramethylsilane (TMS) as the internal standard. The numbering of atoms in the ^{13}C-{^1H} and ^1H NMR spectra of the compounds **2a-h**, **3c**,**e**, **5a-e**, **6a-d** is shown in Figures 1,2,3. Elemental analysis was performed using a Carlo-Erba CHN 1106 elemental analyser. Mass spectra were obtained on a Finnigan 4021 instrument. The yields were calculated from the isolated amount of allylamines obtained from starting 2-alkynylamines.

Preparation of (*Z*)-2-alkenylamines **2a-h**, **3c**,**e** via reduction of substituted 2-alkynylamines via Mg-$NbCl_5$.

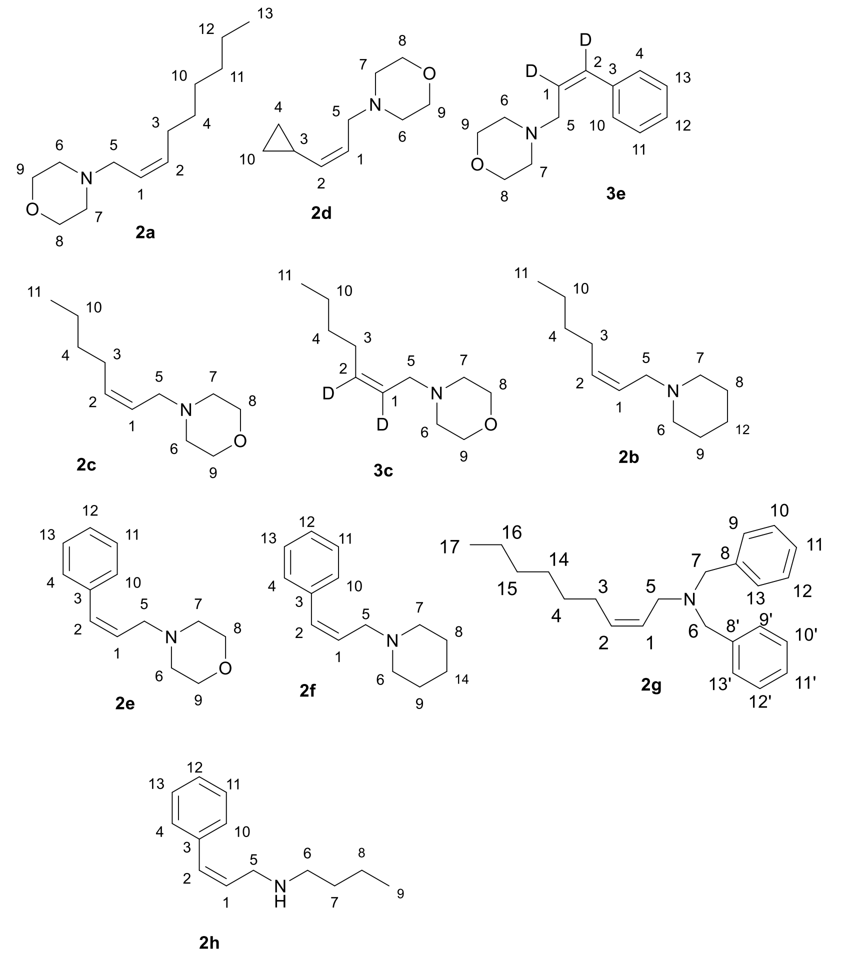

Figure 1. The numbering of atoms in the ^{13}C- and ^{1}H-NMR spectra of the compounds **2a-h**, **3c**,**e**.

(*Z*)-4-(non-2-en-1-yl)morpholine; Typical Procedure.

In a 50-mL reaction flask was placed $NbC1_5$ (2160 mg, 8 mmol) under an argon atmosphere. To the salt was added at room temperature benzene (12 mL) and DME (12 mL) successively. Magnesium powder (144 mg, 6 mmol) was added to the stirring pale yellow solution of $NbC1_5$ and the resulting mixture was stirred at room temperature for 40 min. To the mixture was added at room temperature of a 4-(non-2-yn-1-yl)morpholine (418 mg, 2.0 mmol) and the whole mixture was stirred at 40 °C 7 h. After 7 h at 40 ^{o}C, the reaction mixture was diluted with Et_2O (20 mL), and 25 wt% KOH solution (15 mL) was added dropwise while the reaction flask was cooled in an ice bath. The aqueous layer was extracted with diethyl ether (3×20 mL). The combined organic layers were washed with brine (20 mL), dried over anhydrous $MgSO_4$. The reaction mixture was filtered through a filter paper and concentrated in vacuo to give crude product as a yellow oil. The residue

was distilled through a micro column at 2,4 mmHg to give **2a** (321 mg, 76%) as a colourless oil. b.p. 126 – 127 °C (2,4 mmHg).

^{1}H NMR (500MHz, $CDCl_3$): δ = 0.86 (t, *J* = 6 Hz, 3H, C(13)H_3), 1.22 – 1.28 (m, 6H, C(10 - 12)H_2), 1.29 – 1.32 (m, 2H, C(4)H_2), 2.04 (q, *J* = 7 Hz, 2H, C(3)H_2), 2.43 (s, 4H, C(6, 7)H_2), 2.98 (d, *J* = 7 Hz, 2H, C(5)H_2), 3.69 (t, *J* = 3 Hz 4H, C(8, 9)H_2), 5.39 – 5.44 (m, 1H, C(1)H), 5.52 – 5.57 (m, 1H, C(2)H).

^{13}C NMR (125MHz, $CDCl_3$): δ = 14.04(C(13)), 22.59 (C(12)), 27.49 (C(3)), 28.90 (C(10)), 29.46 (C(4)), 31.68 (C(11)), 55.47 (C(5)), 53.61 (C(6, 7)), 66.99 (C(8, 9)), 125.32 (C(1)), 133.75 (C(2)).

MS (EI): m/z, % = 211 (3) [M^+], 126 (5), 87 (100), 86 (40), 57 (30), 40 (15).

Anal. calcd for $C_{13}H_{25}NO$, (%): C, 73.88; H, 11.92; N, 6.63; Found, %: C, 74.83; H, 11.88; N, 6.57.

(*Z*)-1-(hept-2-en-1-yl)piperidine (2b)

Using the procedure described above 358 mg of *1-(hept-2-yn-1-yl)piperidine* (2 mmol) gave crude product that was distilled through a micro column at 3,4 mmHg to afford **2b** (304 mg, 84%) as a colourless oil. b.p. 107 – 110 °C (3,4 mmHg).

^{1}H NMR (500MHz, $CDCl_3$): δ = 0.90 (t, *J* = 7 Hz, 3H, C(11)H_3), 1.32-1.35 (m, 4H, C(4, 10)H_2), 1.44 (s, 2H, C(12)H_2), 1.59 – 1.63 (m, 4H, C(8, 9)H_2), 2.06 (q, *J* = 6 Hz, 2H, C(3)H_2), 2.42 (s, 4H, C(6,7)H_2), 3.01 (d, *J* = 6 Hz, 2H, C(5)H_2), 5.46 – 5.51 (m, 1H, C(1)H), 5.52 – 5.57 (m, 1H, C(2)H).

^{13}C NMR (125MHz, $CDCl_3$): δ = 13.96 (C(11)), 22.31 (C(10)), 24.26 (C(12)), 25.81 (C(8, 9)), 27.19 (C(3)), 31.72 (C(4)), 54.34 (C(6, 7)), 55.68 (C(5)), 125.91 ((C(1)), 133.15 (C(2)).

MS (EI): m/z, % = 181 (7) [M^+], 138 (4), 124 (10), 98 (29), 84 (100), 55 (30), 41 (15).

Anal. calcd for $C_{12}H_{23}N$, (%): C, 79.49; H, 12.79; N, 7.72; Found, %: C, 79.45; H, 12.83; N, 7.52.

(*Z*)-4-(hept-2-en-1-yl)morpholine (2c)

Using the procedure described above 362 mg of *4-(hept-2-yn-1-yl)morpholine* (2 mmol) gave crude product that was distilled through a micro column at 5 mmHg to afford **2c** (326 mg, 89%) as a colourless oil. b.p. 109 – 111 °C (5 mmHg).

^{1}H NMR (500MHz, $CDCl_3$): δ = 0.82 (t, *J* = 6 Hz, 3H, C(11)H_3), 1.25 – 1.29 (m, 4H, C(4, 10)H_2), 1.99 (q, *J* = 7 Hz, 2H, C(5)H_2), 3.64 (s, 4H, C(8, 9)H_2), 5.35 – 5.39 (m, 1H, C(1)H), 5.47 – 5.52 (m, 1H, C(2)H).

^{13}C NMR (125MHz, $CDCl_3$): δ = 13.85 (C(11)), 22.21 (C(10)), 27.12 (C(3)), 31.61 (C(4)), 55.36 (C(5)), 53.49 (C(6, 7)), 66.83 (C(8, 9)), 125.17 ((C(1)), 133.68 (C(2)).

MS (EI): m/z, % = 183 (10) [M^+], 140 (4), 110 (28), 87 (100), 57 (70), 41 (21).

Anal. calcd for $C_{11}H_{21}NO$, (%): C, 72.08; H, 11.55; N, 7.64; Found, %: C, 72.22; H, 11.56; N, 7.37.

(*Z*)-4-(3-cyclopropylallyl)morpholine (2d)

Using the procedure described above 330 mg of *4-(3-cyclopropylprop-2-yn-1-yl)morpholine* (2 mmol) gave crude product that was distilled through a micro column at 4 mmHg to afford **2d** (267 mg, 80%) as a colourless oil. b.p. 91 – 93 °C (4 mmHg).

^1H NMR (500MHz, $CDCl_3$): δ = 0.29 – 0.32 (m, 2H (A), C(4, 10)H_2), 0.69 – 0.74 (m, 2H (B), C(4, 10)H_2), 1.55 – 1.57 (m, 1H, C(3)H), 2.46 (s, 4H, C(6, 7)H_2), 3.09 (d, *J* = 7 Hz, 2H, C(5)H_2), 3.69 (t, *J* = 4 Hz, 4H, C(8, 9)H_2), 5.32 – 5.37 (m, 1H, C(1)H), 4.89 (t, *J* = 10 Hz, 1H, C(2)H).

^{13}C NMR (125MHz, $CDCl_3$): δ = 6.97 (C(4, 10)), 9.78 (C(3)), 53.62 (C(6, 7)), 55.86 (C(5)), 67.01 (C(8, 9)), 123.39 (C(1)), 137.80 (C(2)).

MS (EI): m/z, % = 167 (10) [M^+], 138 (33), 87 (70), 79 (87), 56 (69), 40 (100).

Anal. calcd for $C_{10}H_{17}NO$, (%): C, 71.81; H, 10.25; N, 8.37; Found, %: C, 71.98; H, 10.35; N, 8.35.

(*Z*)-4-(3-phenylallyl)morpholine (2e)

Using the procedure described above 330 mg of 402 mg of *4-(3-phenylprop-2-yn-1-yl)morpholine* (2 mmol) gave crude product that was distilled through a micro column at 1 mmHg to afford **2e** (305 mg, 75%) as a colourless oil. b.p. 130 – 132 °C (1 mmHg).

^1H NMR (500MHz, $CDCl_3$): δ = 2.49 (s, 4H, C(6, 7)H_2), 3.29-3.31(dd, J = 2 Hz, *J* = 6 Hz, 2H, C(5)H_2), 3.75 (t, *J* = 4 Hz, 4H, C(8, 9)H_2), 5.78 – 5.83 (m, 1H, C(1)H), 6.63 (d, *J* = 11 Hz, 1H, C(2)H), 7.26 – 7.29 (m, 3H, C(4, 10, 12)H), 7.37 (t, *J* = 8 Hz, 2H, C(11, 13)H).

^{13}C NMR (125MHz, $CDCl_3$): δ = 53.71 (C(6, 7)), 59.05 (C(5)), 67.03 (C(8, 9)), 126.97 (C(12)), 128.17 (C(11, 13)), 128.89 (C(4, 10)), 129.00 (C(1)), 131.69 (C(2)), 137.00 (C(3)).

MS (EI): m/z, % = 203 (20) [M^+], 172 (4), 144 (12), 117 (72), 112 (100), 91 (33), 56 (32).

Anal. calcd for $C_{13}H_{17}NO$, (%): C, 76.81; H, 8.43; N, 6.89; O, 7.87; Found, %: C, 76.90; H, 8.37; N, 7.01.

(*Z*)-1-(3-phenylallyl)piperidine (2f)

Using the procedure described above 398 mg of *1-(3-phenylprop-2-yn-1-yl)piperidine* (2 mmol) gave crude product that was distilled through a micro column at 1 mmHg to afford **2f** (293 mg, 73%) as a colourless oil. b.p. 122 – 124 °C (1 mmHg).

^{1}H NMR (500MHz, $CDCl_3$): δ = 1.45 (C(14)), 1.59 – 1.64 (m, 4H, C(8, 9)H_2), 2.42 (s, 4H, C(6, 7)H_2), 3.28 (d, *J* = 6 Hz, 2H, C(5)H_2), 5.82 – 5.87 (m, 1H, C(1)H), 6.57 (d, *J* = 12 Hz, 1H, C(2)H), 7.24 – 7.28 (m, 3H, C(4, 10, 12)H), 7.33 – 7.37 (m, 2H, C(11, 13)H).

^{13}C NMR (125MHz, $CDCl_3$): δ = 24.29 (C(14)), 26.01 (C(8,9)), 54.70 (C(6, 7)), 57.12 (C(5)), 126.75 (C(12)), 128.09 (C(11, 13)), 128.91 (C(4, 10)), 130.31 (C(1)), 130.75 (C(2)), 137.29 (C(3)).

MS (EI): m/z, % = 201 (12) [M^+], 200 (15), 117 (44), 115 (38), 110 (100), 98 (30), 84 (12).

Anal. calcd for $C_{14}H_{19}N$, (%): C, 83.53; H, 9.51; N, 6.96; Found, %: C, 83.61; H, 9.47; N, 7.12.

(*Z*)-4-(hept-2-en-1-yl-2,3-*d*$_2$)morpholine (3c)

Using the procedure described above 362 mg of *4-(hept-2-yn-1-yl)morpholine* (2 mmol) and D_2O gave crude product that was distilled through a micro column at 2,4 mmHg to afford **3c** (303 mg, 82%) as a colourless oil. b.p. 118 – 120 °C (2,4 mmHg). IR (liquid film): 2958, 2925, 2870, 2855, 2811, 1742, 1742, 1654, 1618, 1519, 1508, 1456, 1399, 1118, 1070, 1034, 1008, 943, 866, 801 cm^{-1}.

^{1}H NMR (500MHz, $CDCl_3$): δ = 0.91 (t, *J* = 6 Hz, 3H, C(11)H_3), 1.34 – 1.38 (m, 4H, C(4, 10)H_2), 2.08 (t, *J* = 6 Hz, 2H, C(3)H_2), 2.47 (s, 4H, C(6, 7)H_2), 3.03 (s, 2H, C(5)H_2), 3.74 (s, 4H, C(8, 9)H_2).

^{13}C NMR (125MHz, $CDCl_3$): δ = 13.96 (C(11)), 22.32 (C(10)), 27.09 (C(3)), 31.69 (C(4)), 53.61 (C(6, 7)), 55.36 (C(5)), 67.01 (C(8, 9)).

MS (EI): m/z, % = 185 (7) [M^+], 156 (1), 128 (6), 112 (19), 87 (100), 57 (70), 57 (70), 42 (13).

Anal. calcd for $C_{11}H_{19}D_2NO$, (%): C, 71.30; N, 7.56; Found, %: C, 71.46; N, 7.42.

(*Z*)-4-(3-phenylallyl-2,3-*d*$_2$)morpholine (3e)

Using the procedure described above 402 mg of *4-(3-phenylprop-2-yn-1-yl)morpholine* (2 mmol) and D_2O gave crude product that was distilled through a micro column at 1 mmHg to afford **3e** (316 mg, 77%) as a colourless oil. b.p. 130 – 132 °C (1 mmHg). IR (liquid film): 3021, 2959, 2926, 2855, 2807, 2759, 1519, 1493, 1454, 1316, 1294, 1216, 1117, 1007, 778, 700, 669, 598, 472 cm^{-1}.

^{1}H NMR (500MHz, $CDCl_3$): δ = 2.49 (s, 4H, C(6, 7)H_2), 3.29 (s, 2H, C(5)H_2), 3.74 (t, *J* = 4 Hz, 4H, C(8, 9)H_2), 7.26 – 7.29 (m, 3H, C(4, 10, 12)H), 7.37 (t, *J* = 8 Hz, 2H, C(11, 13)H).

^{13}C NMR (125MHz, $CDCl_3$): δ = 53.72 (C(6, 7)), 56.51 (C(5)), 67.02 (C(8, 9)), 126.97 (C(12)), 128.17 (C(11, 13)), 128.89 (C(4, 10)), 136.93 (C(3)).

MS (EI): m/z, % = 205 (21) [M^+], 204 (14), 146 (9), 119 (73), 113 (100), 86 (19), 56 (32).

Anal. calcd for $C_{13}H_{15}D_2NO$, (%): C, 76.06; N, 6.82. Found, %: C, 76.13; N, 6.95.

(*Z*)-*N*,*N*-dibenzylnon-2-en-1-amine (**2g**)

Using the procedure described above 638 mg of *N,N-dibenzylnon-2-yn-1-amine* (2 mmol) and H_2O gave crude product that was distilled through a micro column at 1 mmHg to afford **2g** (567 mg, 88%) as a colourless oil. b.p. 193 – 195 °C (1 mmHg).

^{1}H NMR (500MHz, CDCl3): δ = 0.97 (t, *J* = 4 Hz, 3H, C(17)H_3), 1.33 – 1.43 (m, 8H, C(4, 5, 14, 15,)H_2), 2.04 – 2.08 (m, 2H, C(3)H_2), 3.14 – 3.16 (m, 2H, C(5)H_2), 3.64 (s, 4H, C(6, 7)H_2), 5.62 – 5.65 (m, 2H, C(1, 2)H), 7.28 – 7.32 (m, 2H, C(11, 11')H), 7.37 - 7.39 (m, 4H, C(10, 12, 10', 12')H), 7.44 – 7.48 (m, 4H, C(9, 13, 9', 13')H).

^{13}C NMR (125MHz, CDCl3): δ = 14.18 (C(17)), 22.71 (C(16)), 27.65 (C(3)), 29.04 (C(4)), 29.66 (C(14)), 31.80 (C(15)), 50.13 (C(5)), 58.03 (2C(6, 7)), 126.82 (3C (1, 11, 11')), 128.19 (4C (10, 12, 10', 12'), 128.87 (4C (9, 13, 9', 13'), 133.22 (C(2)), 139.88 (2C (8, 8')).

MS (EI): m/z, % = 322 (<1) [M^+], 232 (3), 210 (59), 181 (4), 91 (100), 65 (4).

Anal. calcd for $C_{23}H_{31}N$, (%): C, 85.92; H, 9.72; N, 4.36. Found, %: C, 86.86; H, 9.68; N, 4.10.

(*Z*)-*N*-(3-phenylallyl)butan-1-amine (**2h**)

Using the procedure described above 374 mg of *N-(3-phenylprop-2-yn-1-yl)butan-1-amine* (2 mmol) and H_2O gave crude product that was distilled through a micro column at 3 mmHg to afford **2h** (336 mg, 89%) as a colourless oil. b.p. 130 – 132 °C (3 mmHg).

^{1}H NMR (500MHz, $CDCl_3$): δ = 0.93 (t, *J* = 7 Hz, 3H, C(9)H_3), 1.32 – 1.39 (m, 2H, C(8)H_2), 1.46– 1.52 (m, 2H, C(7)H_2), 2.65 (t, *J* =7 Hz, 2H, C(6)H_2), 3.57 (d, *J* =6 Hz, 2H, C(5)H_2), 5.78 – 5.83 (m, 1H, C(1)H), 6.55 (d, *J* = 12 Hz, 1H, C(2)H), 7.25 – 7.28 (m, 3H, C(4, 10, 12)H), 7.34 – 7.37 (t, *J* = 7 Hz, 2H, C(11, 13)H).

^{13}C NMR (125MHz, $CDCl_3$): δ = 13.99 (C(9)), 20.48 (C(8)), 32.14 (C(7)), 47.71(C(5)), 49.34 (C(6)), 126.89 (C(12)), 128.17 (2C(11, 13)), 128.78 (2C(4, 10)), 130.41 (C(2)), 131.15 (C(1), 137.12 (C(3)).

MS (EI): m/z, % = 189 (7) [M^+], 146 (10), 117 (100), 91 (14), 84 (10).

Anal. calcd for $C_{13}H_{19}N$, (%): C, 82.48; H, 10.12; N, 7.40. Found, %: C, 82.35; H, 10.01; N, 7.33.

Preparation of (3*Z*)-alkenylols **5a-e** via reduction of substituted alkynylols via Mg-$NbCl_5$.

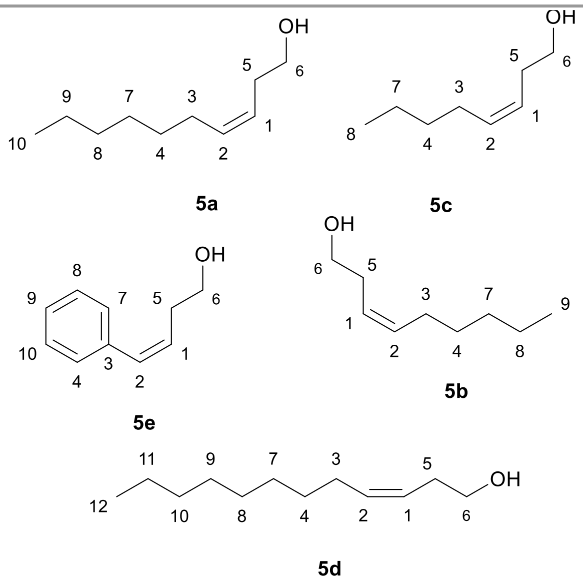

Figure 2. The numbering of atoms in the ^{13}C- and ^1H-NMR spectra of the compounds **5a-e.**

(*Z*)-dec-3-en-1-ol; Typical Procedure.

In a 50-mL reaction flask was placed $NbC1_5$ (2160 mg, 8 mmol) under an argon atmosphere. To the salt was added at room temperature benzene (12 mL) and DME (12 mL) successively. Magnesium powder (144 mg, 6 mmol) was added to the stirring pale yellow solution of $NbC1_5$ and the resulting mixture was stirred at room temperature for 40 min. To the mixture was added at room temperature of a dec-3-yn-1-ol (616 mg, 3.0 mmol) and the whole mixture was stirred at room temperature 8 h. After 8 h at room temperature, the reaction mixture was diluted with Et_2O (20 mL), and 25 wt% KOH solution (15 mL) was added dropwise while the reaction flask was cooled in an ice bath. The aqueous layer was extracted with diethyl ether (3×20 mL). The combined organic layers were washed with brine (20 mL), dried over anhydrous $MgSO_4$. The reaction mixture was filtered through a filter paper and concentrated in vacuo to give crude product as a yellow oil. The

residue was distilled through a micro column at 4 mmHg to give **5a** (275 mg, 88%) as a colourless oil. b.p. 99 – 101 °C (4 mmHg).

^{1}H NMR (500MHz, $CDCl_3$): δ = 0.90 (t, *J* = 6 Hz, 3H, C(10)H_3), 1.27 – 1.29 (m, 8H, C(4, 7, 8, 9)H_2), 2.04 – 2.06 (m, 2H, C(3)H_2), 2.35 (q, *J* = 7 Hz, 2H, C(5)H_2), 3.66 (t, *J* = 6 Hz, 2H, C(6)H_2), 5.36 – 5.41 (m, 1H, C(1)H), 5.56 – 5.61 (m, 1H, C(2)H).

^{13}C NMR (125MHz, $CDCl_3$): δ = 14.09 (C(10)), 22.63 (C(9)), 27.38 (C(3)), 29.67 (C(4)), 29.70 (C(7)), 30.90 (C(5)), 31.75 (C(8)), 62.37 (C(6)), 124.93 (C(1)), 133.58 (C(2)).

MS (EI): m/z, % = 170 (3) [M^+], 111 (11), 95 (25), 83 (47), 69 (74), 55 (100), 41 (46).

Anal. calcd for $C_{10}H_{20}O$, (%): C, 76.86; H, 12.90; Found, %: C, 77.04; H, 12.83.

(*Z*)-non-3-en-1-ol (5b)

Using the procedure described above 280 mg of *non-3-yn-1-ol* (2 mmol) gave crude product that was distilled through a micro column at 10 mmHg to afford **5b** (256 mg, 90%) as a colourless oil. b.p. 93 – 95 °C (10 mmHg). The spectral properties (^{1}H NMR, ^{13}C NMR, MS) were in good agreement with those that were reported in the literature [3].

(*Z*)-oct-3-en-1-ol (5c)

Using the procedure described above 252 mg of *oct-3-yn-1-ol* (2 mmol) gave crude product that was distilled through a micro column at 14 mmHg to afford **5c** (210 mg, 82%) as a colourless oil. b.p. 88 – 90 °C (14 mmHg).

^{1}H NMR (500MHz, $CDCl_3$): δ = 0.93 (t, *J* = 7 Hz, 3H, C(8)H_3), 1.39 – 1.44 (m, 2H, C(7)H_2), 1.47 – 1.53 (m, 2H, C(4)H_2), 2.19 (t, *J* = 7 Hz, 2H, C(3)H_2), 2.44 – 2.47 (m, 2H, C(5)H_2), 3.69 (br. s, 2H, C(6)H_2), 5.36 – 5.40 (m, 2H, C(1)H), 5.53 – 5.61 (m, 1H, C(2)H).

^{13}C NMR (125MHz, $CDCl_3$): δ = 13.61 (C(8)), 18.92 (C(3)), 21.96 (C(7)), 23.19 (C(5)), 31.07 (C(4)), 61.39 (C(6)), 124.93 (C(1)), 133.56 (C(2)).

Anal. calcd for $C_8H_{16}O$, (%): C, 74.94; H, 12.58; Found, %: C, 75.12; H, 12.67.

(*Z*)-dodec-3-en-1-ol (5d)

Using the procedure described above 362 mg of *dodec-3-yn-1-ol* (2 mmol) gave crude product that was distilled through a micro column at 2 mmHg to afford **5d** (339 mg, 92%) as a colourless oil. b.p. 115 – 117 °C (2 mmHg). The spectral properties (^{1}H NMR, ^{13}C NMR, MS) were in good agreement with those that were reported in the literature [4].

(*Z*)-4-phenylbut-3-en-1-ol (5e)

Using the procedure described above 292 mg of *4-phenylbut-3-yn-1-ol* (2 mmol) gave crude product that was distilled through a micro column at 1.8 mmHg to afford **5e** (216 mg, 73%) as a colourless oil. b.p. 117 – 121 °C (1.8 mmHg).

^{1}H NMR (500MHz, $CDCl_3$): δ = 2.64 (q, *J* = 7 Hz, 2H, C(5)H_2), 3.74 (t, *J* = 6 Hz, 2H, C(6)H_2), 5.71 – 5.76 (m, 1H, C(1)H), 6.61 (d, *J* = 12 Hz, 1H, C(2)H), 7.23 – 7.29 (m, 1H, C(9)H), 7.33 – 7.39 (m, 4H, C(4, 7, 8, 10)H).

^{13}C NMR (125MHz, $CDCl_3$): δ = 32.02 (C(5)), 62.37 (C(6)), 126.83 (C(8)), 128.46 (C(1)), 131.36 (C(2)), 128.26 (C(8, 10)), 128.78 (C(4, 7)), 137.32 (C(3)).

MS (EI): m/z, % = 148 (40) [M^+], 117 (100), 115 (95), 104 (89), 91 (66), 77 (9), 65 (15).

Anal. calcd for $C_{10}H_{12}O$ (%): C, 81.04; H, 8.16. Found, %: C, 80.73; H, 8.19.

Preparation of (3*Z*)-alkenylols **6a-d** via chlorothiolation of alkynes with the $NbCl_5$–Mg reagent system.

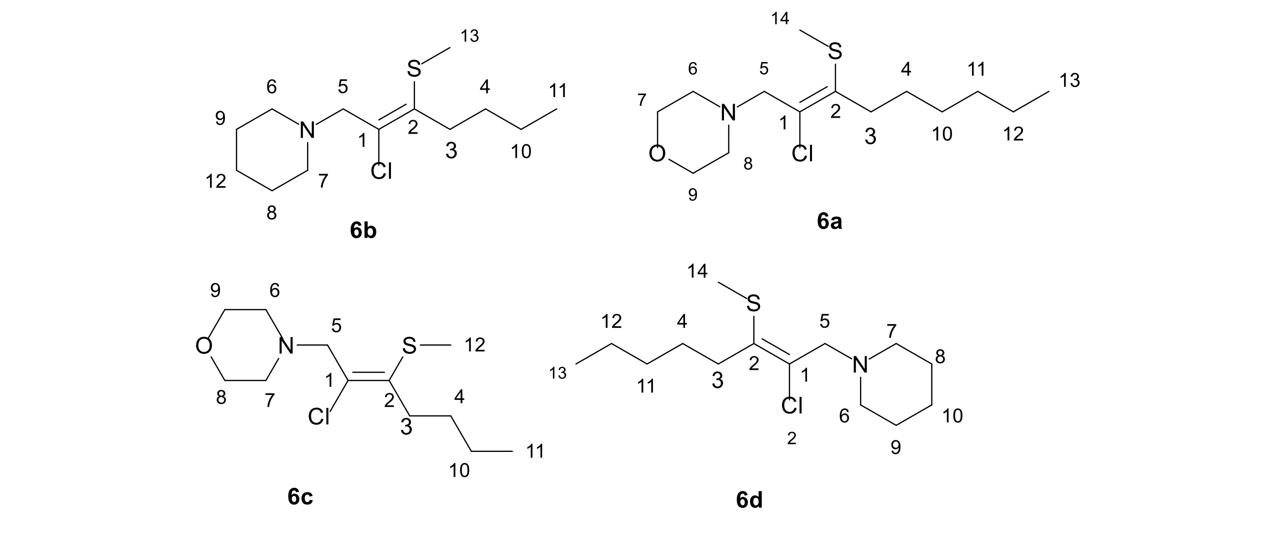

Figure 3. The numbering of atoms in the ^{13}C- and ^{1}H-NMR spectra of the compounds **6a-d.**

(*E*)-4-(2-chloro-3-(methylthio)non-2-en-1-yl)morpholine; Typical Procedure.

In a 50-mL reaction flask was placed $NbC1_5$ (2160 mg, 8 mmol) under an argon atmosphere. To the salt was added at room temperature benzene (12 mL) and DME (12 mL) successively. Magnesium powder (144 mg, 6 mmol) and a 4-(non-2-yn-1-yl)morpholine (418 mg, 2.0 mmol) were added to the stirring pale yellow solution of $NbC1_5$ the resulting mixture was stirred at 40 °C for 4 h. After 4 h at 40 °C to the mixture was added at room temperature of a methanesulfonyl chloride (690 mg, 6.0 mmol) and the whole mixture was stirred at room temperature 8 h. After 8 h at room temperature, the

reaction mixture was diluted with Et_2O (20 mL), and 25 wt% KOH solution (15 mL) was added dropwise while the reaction flask was cooled in an ice bath. The aqueous layer was extracted with diethyl ether (3×20 mL). The combined organic layers were washed with brine (20 mL), dried over anhydrous $MgSO_4$. The reaction mixture was filtered through a filter paper and concentrated in vacuo to give crude product as a yellow oil. The residue was distilled through a micro column at 1.6 mmHg to give **6a** (456 mg, 78%) as a colourless oil. b.p. 97 – 100 ºC (1.6 mmHg).

^{1}H NMR (500MHz, $CDCl_3$): δ = 0.91 (t, *J* = 7 Hz, 3H, C(13)H_3), 1.33 (s, 6H, C(12, 11, 10)H2), 1.56-1.60 (m, 2H, C(4)), 2.36 (s, 3H, C(14)H_3), 2.52 (t, *J* = 7 Hz, 4H, C(6, 7)H_2), 2.74 (t, *J* = 7 Hz, 2H, C(3)H_2), 3.42 (s, 2H, C(5)H_2), 3.73 (t, *J* = 4 Hz, C(8, 9)).

^{13}C NMR (125MHz, $CDCl_3$): δ = 14.05 (C(13)), 16.35 (C(14)), 22.57 (C(12)), 27.49 (C(4)), 28.50 (C(10)), 31.58 (C(11)), 37.23(C(3)), 53.19 (C(6, 7)), 59.09 (C(5)), 67.08 (C(8, 9)), 127.42 (C(2)), 136.95(C(1))MS (EI): m/z, % = 292 (<1) [M^+], 291 (2), 189 (<1), 100 (100), 86 (7).

Anal. calcd for $C_{14}H_{26}ClNOS$, (%): C, 57.41; H, 8.98; N, 4.80. Found, %: C, 57.74; H, 8.83; N, 4.89.

(*E*)-1-(2-chloro-3-(methylthio)hept-2-en-1-yl)piperidine (6b)

Using the procedure described above 358 mg of *1-(hept-2-yn-1-yl)piperidine* (2 mmol) gave crude product that was distilled through a micro column at 2 mmHg to afford **6b** (419 mg, 80%) as a colourless oil. b.p. 97 – 99 ºC (2 mmHg).

^{1}H NMR (500MHz, $CDCl_3$): δ = 0.96 (t, *J* = 7 Hz, 3H, C(11)H_3), 1.37 (q, *J* = 7 Hz, 2H, C(10)H_2), 1.44 – 1.47 (m, 2H, C(12)), 1.55 – 1.61 (m, 6H, C(4, 8, 9)H_2), 2.36 (s, 3H, C(13)H_3), 2.43 (s, 4H, C(6, 7)H_2), 2.74 (t, *J* = 7 Hz, 2H, C(3)H_2), 3.36 (s, 2H, C(5)H_2).

^{13}C NMR (125MHz, $CDCl_3$): δ = 13.95 (C(11)), 16.35 (C(13)), 22.02 (C(10)), 24.45 (C(12)), 26.06 (C(8, 9)), 29.72 (C(4)), 36.98 (C(3)), 54.13 (C(6, 7)), 59.63 (C(5)), 128.52 (C(2)), 135.89 (C(1)).

MS (EI): m/z, % = 262 (1) [M^+], 261 (4), 178 (1), 98 (100), 84 (12).

Anal. calcd for $C_{13}H_{24}ClNS$, (%): C, 59.63; H, 9.24; N, 5.35. Found, %: C, 59.78; H, 9.41; N, 5.59.

(*E*)-4-(2-chloro-3-(methylthio)hept-2-en-1-yl)morpholine (6c)

Using the procedure described above 362 mg of *4-(hept-2-yn-1-yl)morpholine* (2 mmol) gave crude product that was distilled through a micro column at 1.9 mmHg to afford **6c** (375 mg, 71%) as a colourless oil. b.p. 128 – 130 ^oC (1.9 mmHg).

^1H NMR (500MHz, $CDCl_3$): δ = 0.96 (t, *J* = 7 Hz, 3H, $C(11)H_3$), 1.34 – 1.39 (m, 2H, $C(10)H_2$), 1.55 – 1.61 (m, 2H, $C(4)H_2$), 2.36 (s, 3H, $C(12)H_3$), 2.52 (s, 4H, $C(6, 7)H_2$), 2.75 (t, *J* = 7 Hz, 2H, $C(3)H_2$), 3.42 (s, 2H, $C(5)H_2$), 3.74 (s, 4H, $C(8, 9)H_2$).

^{13}C NMR (125MHz, $CDCl_3$): δ = 13.92 (C(11)), 16.35 (C(12)), 22.00 (C(10)), 29.69 (C(4)), 36.99 (C(3)), 53.17 (C(6, 7)), 59.07 (C(5)), 67.06 (C(8, 9)), 127.41 (C(2)), 136.89 (C(1)).

MS (EI): m/z, % = 264 (13) [M^+], 263 (65), 230 (18), 228 (19), 176 (10), 101 (98), 100 (100), 86 (47), 56 (29).

Anal. calcd for $C_{12}H_{22}ClNOS$, (%): C, 54.63; H, 8.41; N, 5.31. Found, %: C, 54.78; H, 8.21; N, 5.29.

Acknowledgements

This work was financially supported by the Russian Science Foundation (grant No. 19-73-10113).

References

1. Bieber, L. W.; da Silva, M. F. Tetrahedron Lett. 2004, 45, 8281 - 8283.
2. Knight, J. A.; Diamond, J. H. Journal of Organic Chemistry 1959, 24, 400–3.
3. Brenna, E.; Crotti, M.; Gatti, F. G.; Marinoni, L.; Monti, D.; Quaiato, S. Journal of Organic Chemistry 2017, 82, 2114–2122.
4. Mack, D. J.; Guoa, B.; Njardarson, J. T., Chem. Commun. 2012, 48, 7844–7846.

^{13}C-NMR spectrum of (*Z*)-4-(non-2-en-1-yl)morpholine (2a)

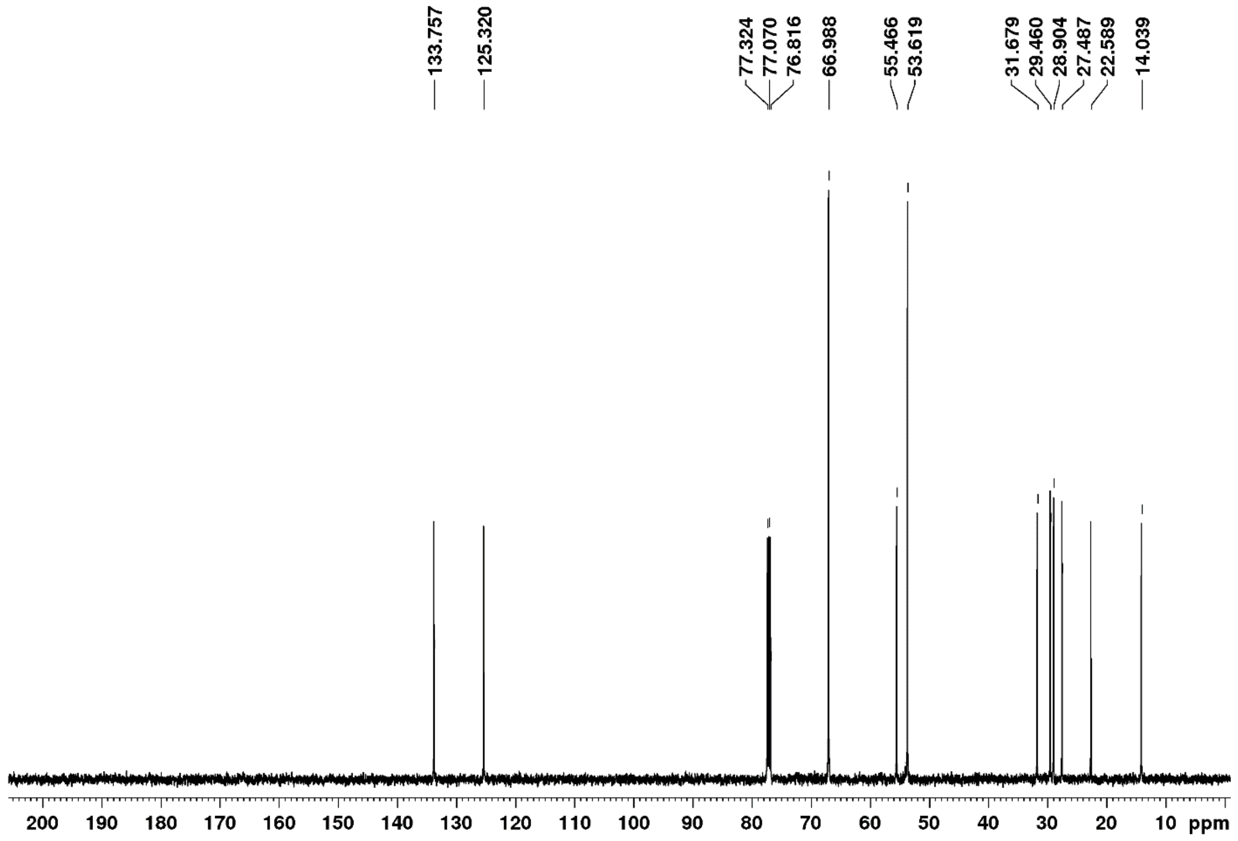

^{1}H-NMR spectrum of (Z)-4-(non-2-en-1-yl)morpholine (2a)

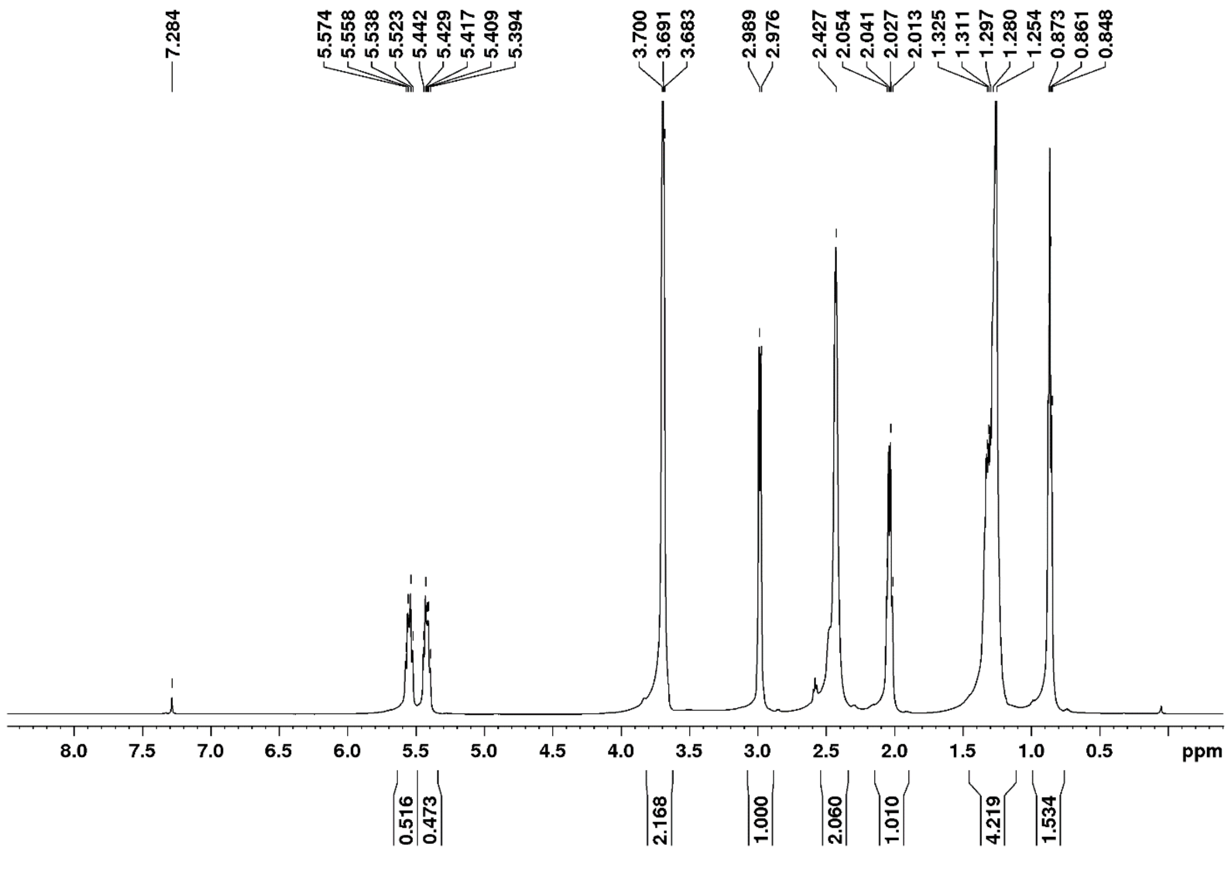

^{13}C-NMR spectrum of (*Z*)-1-(hept-2-en-1-yl)piperidine (2b)

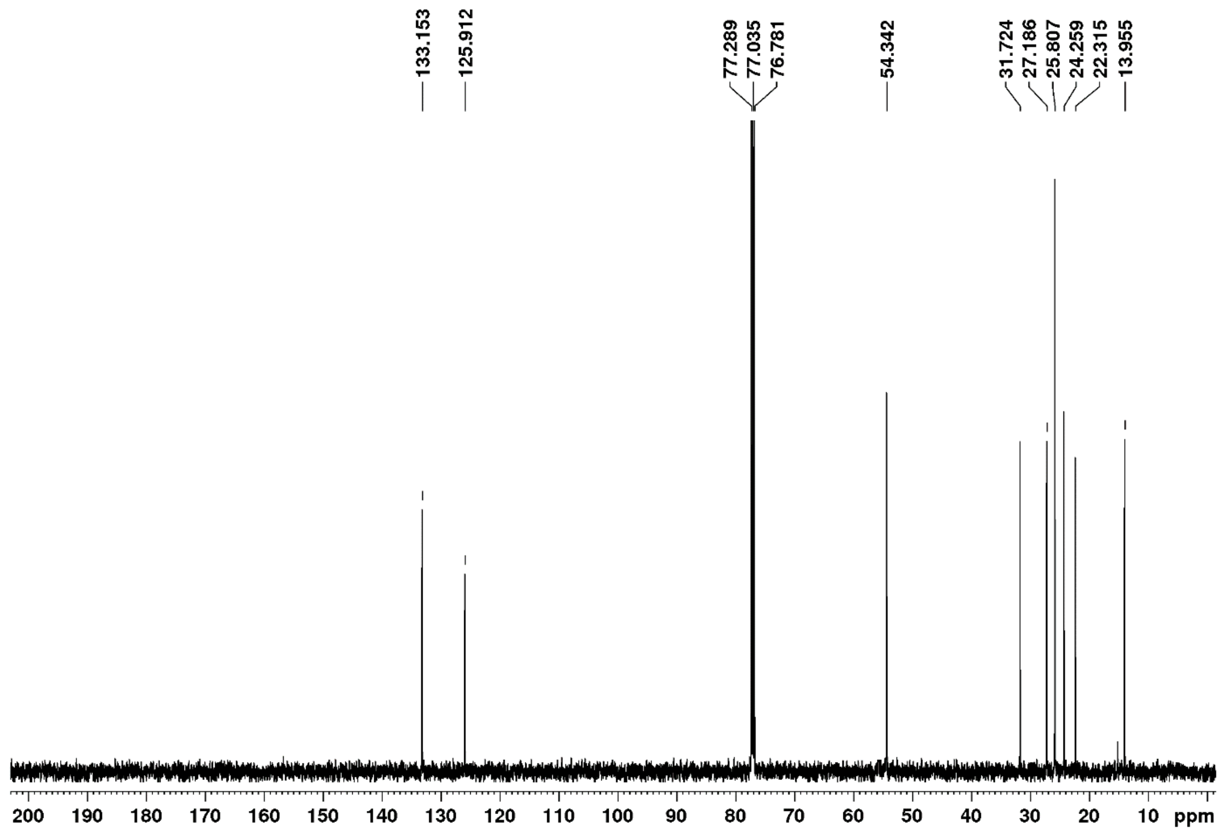

^{1}H-NMR spectrum of (*Z*)-1-(hept-2-en-1-yl)piperidine (2b)

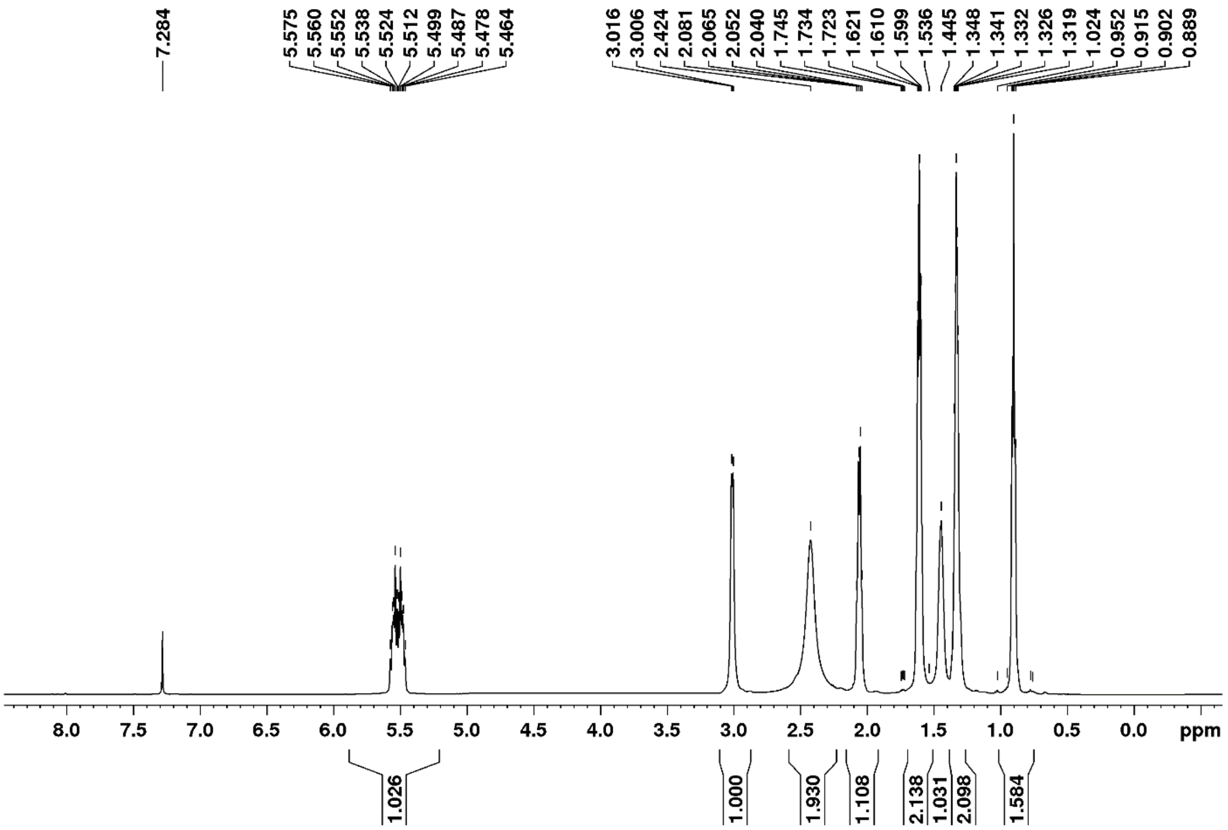

^{13}C-NMR spectrum of (Z)-4-(hept-2-en-1-yl)morpholine (2c)

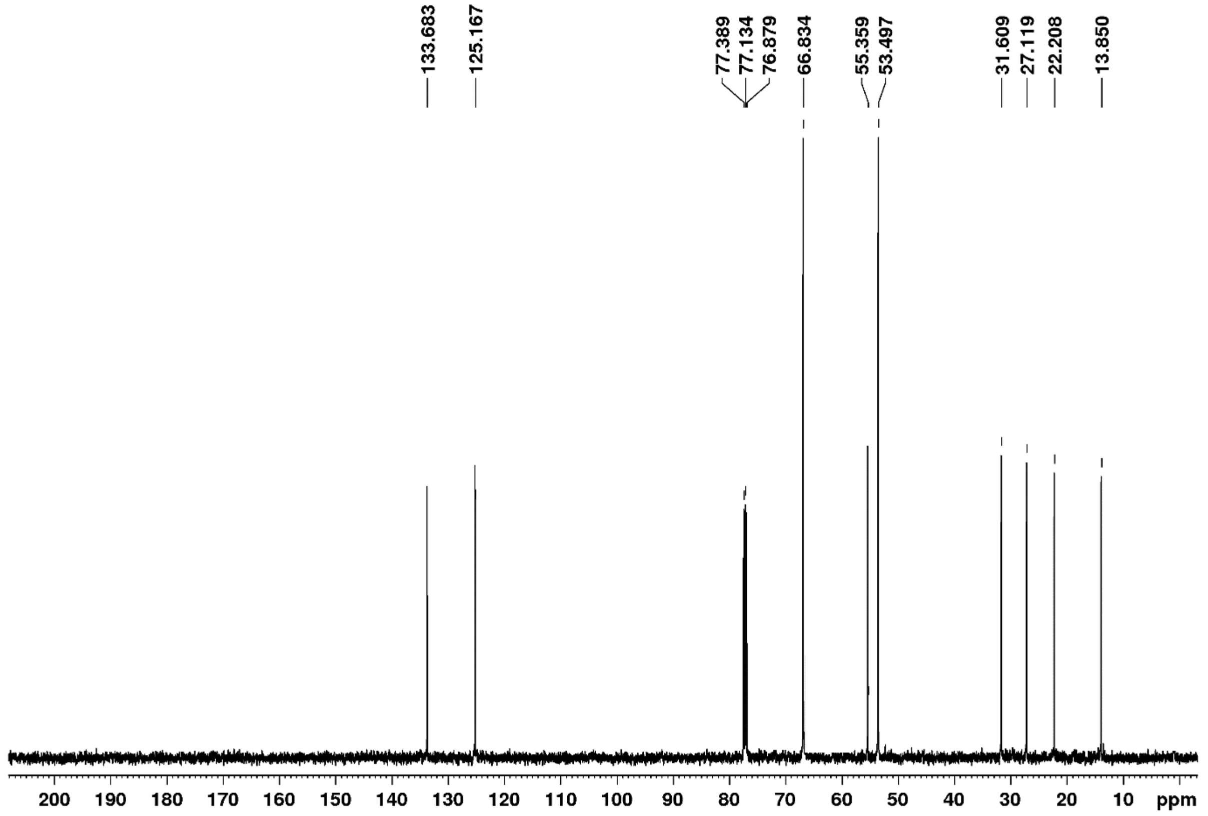

^{1}H-NMR spectrum of (Z)-4-(hept-2-en-1-yl)morpholine (2c)

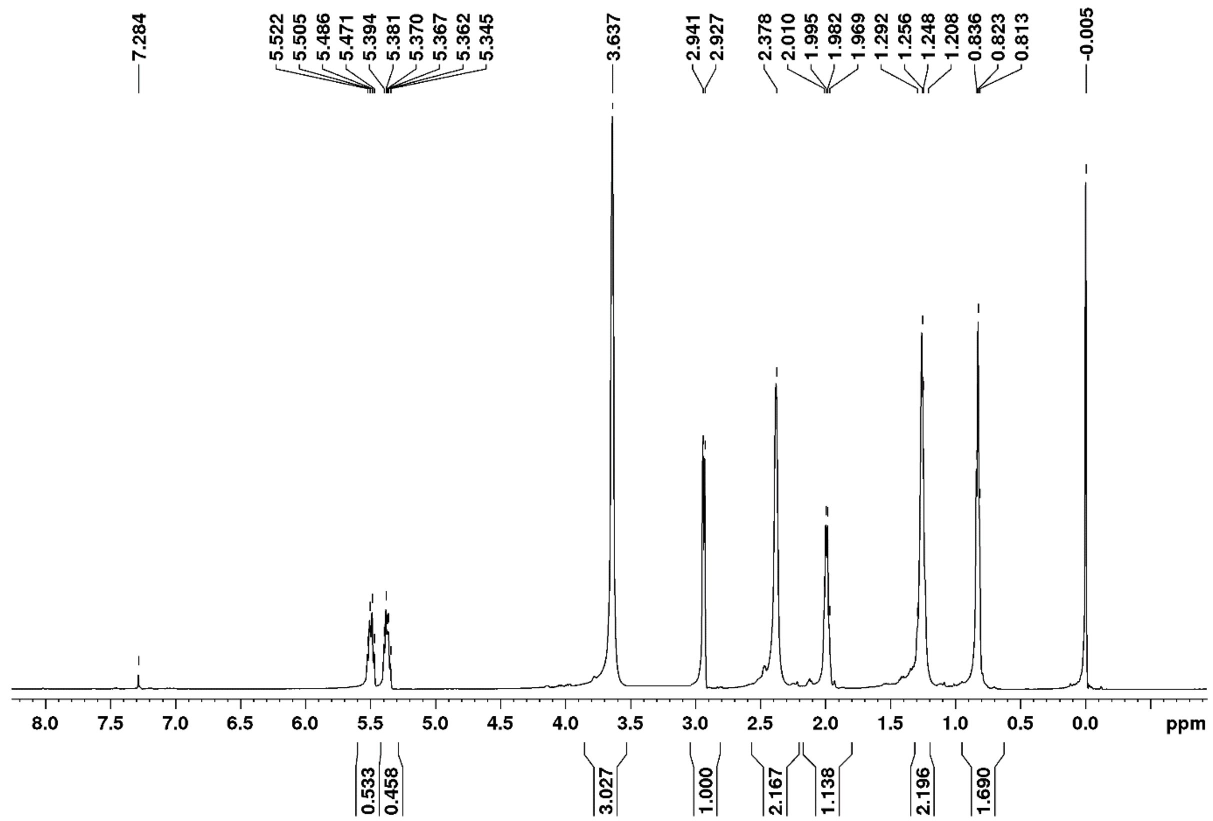

^{13}C-NMR spectrum of (*Z*)-4-(3-cyclopropylallyl)morpholine (2d)

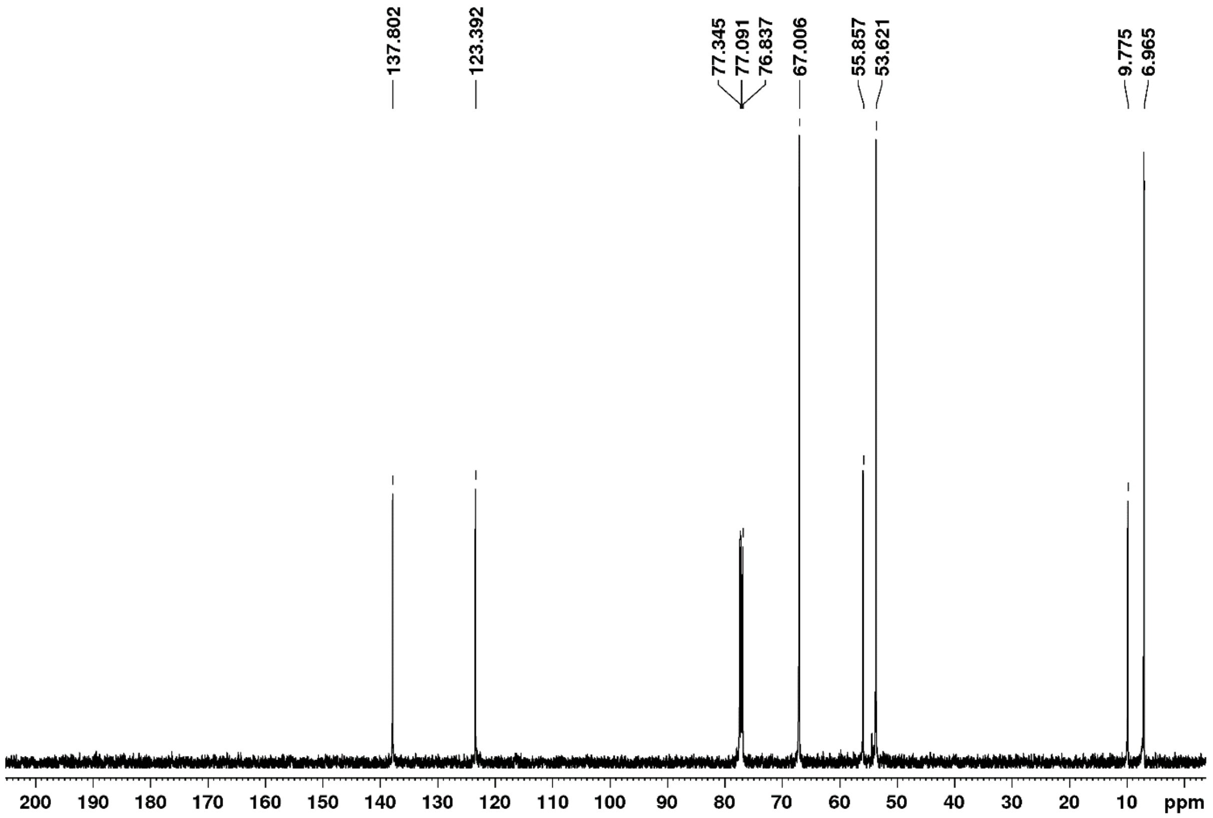

^{1}H-NMR spectrum of (*Z*)-4-(3-cyclopropylallyl)morpholine (2d)

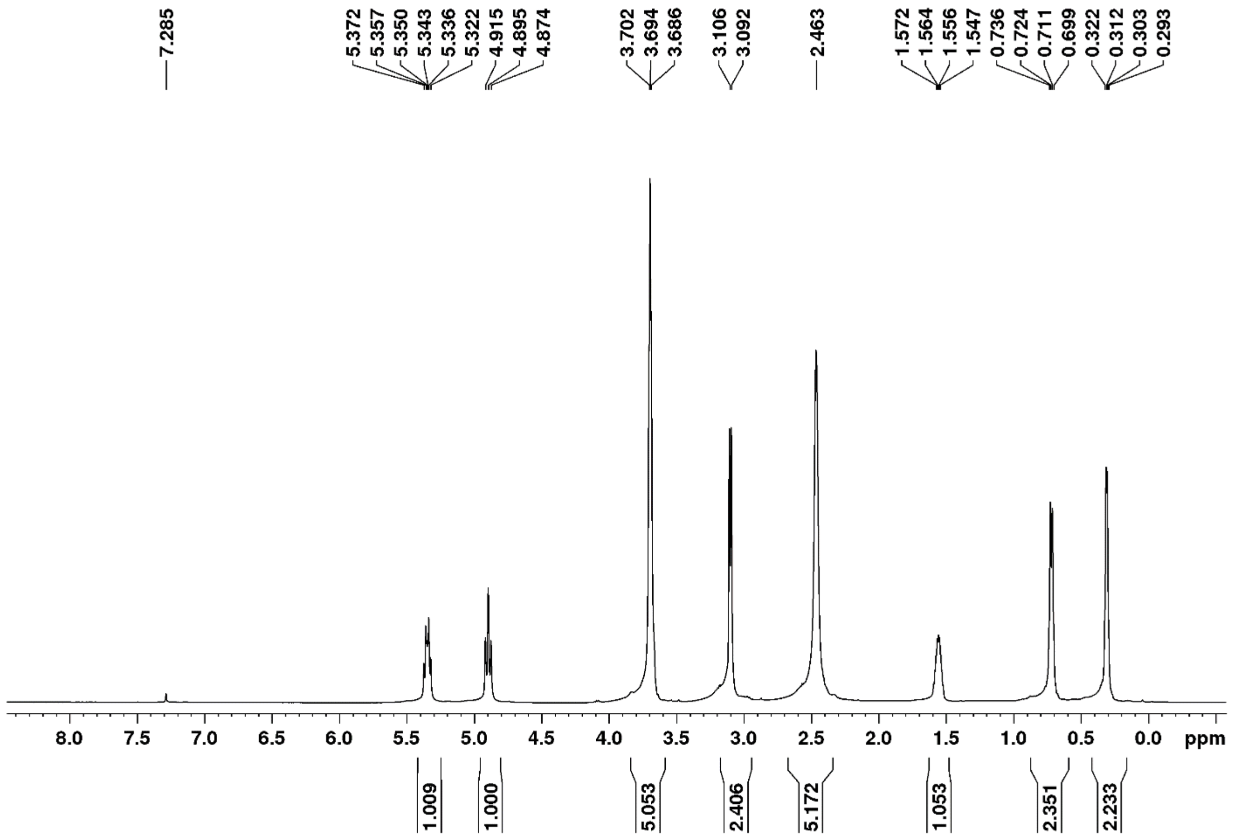

^{13}C-NMR spectrum of (*Z*)-4-(3-phenylallyl)morpholine (2e)

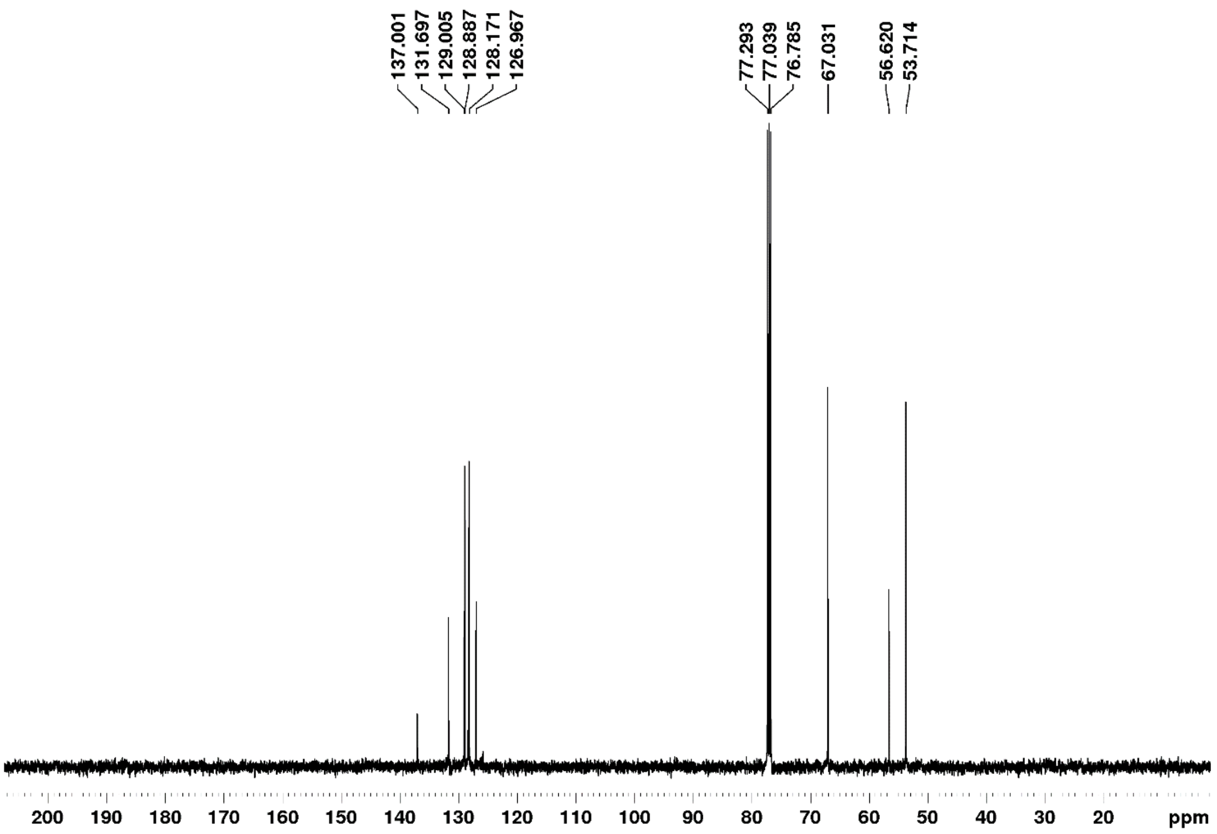

^{1}H-NMR spectrum of (*Z*)-4-(3-phenylallyl)morpholine (2e)

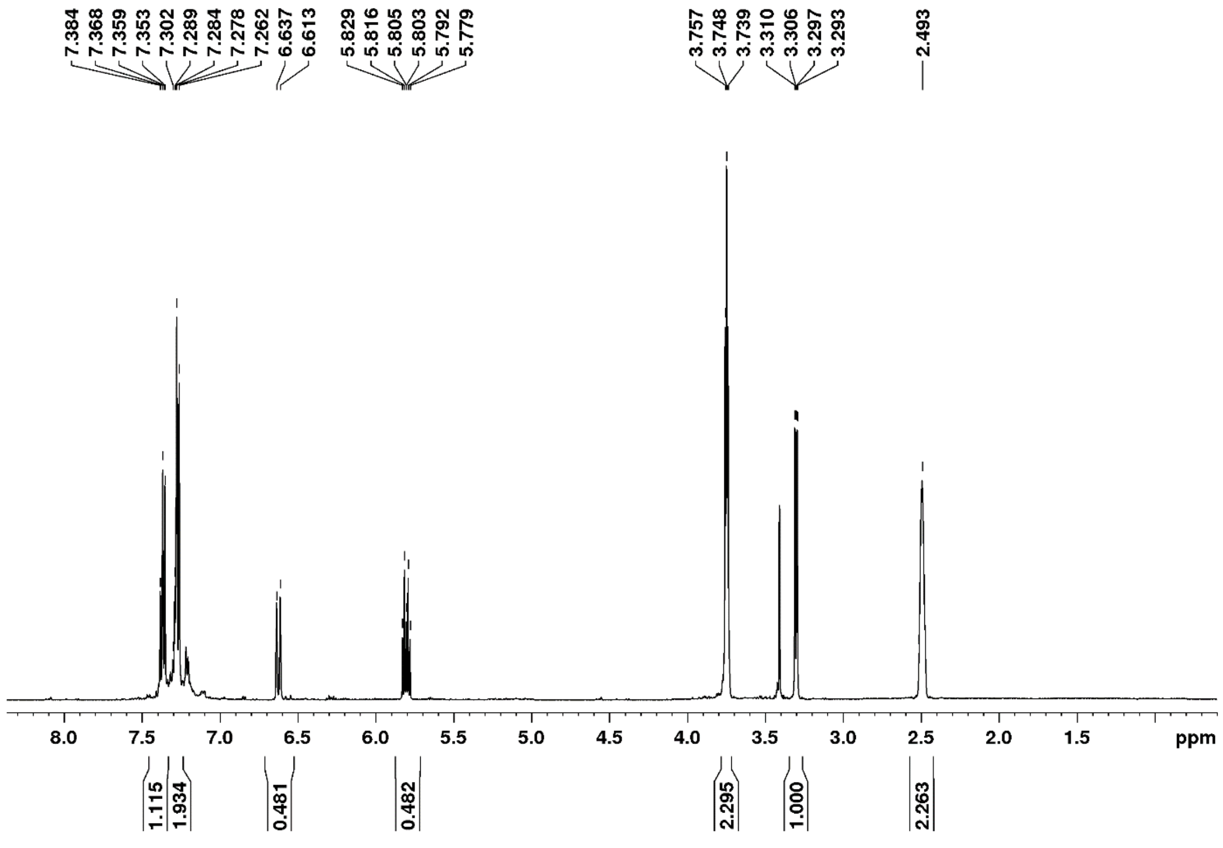

^{13}C-NMR spectrum of (Z)-1-(3-phenylallyl)piperidine (2f)

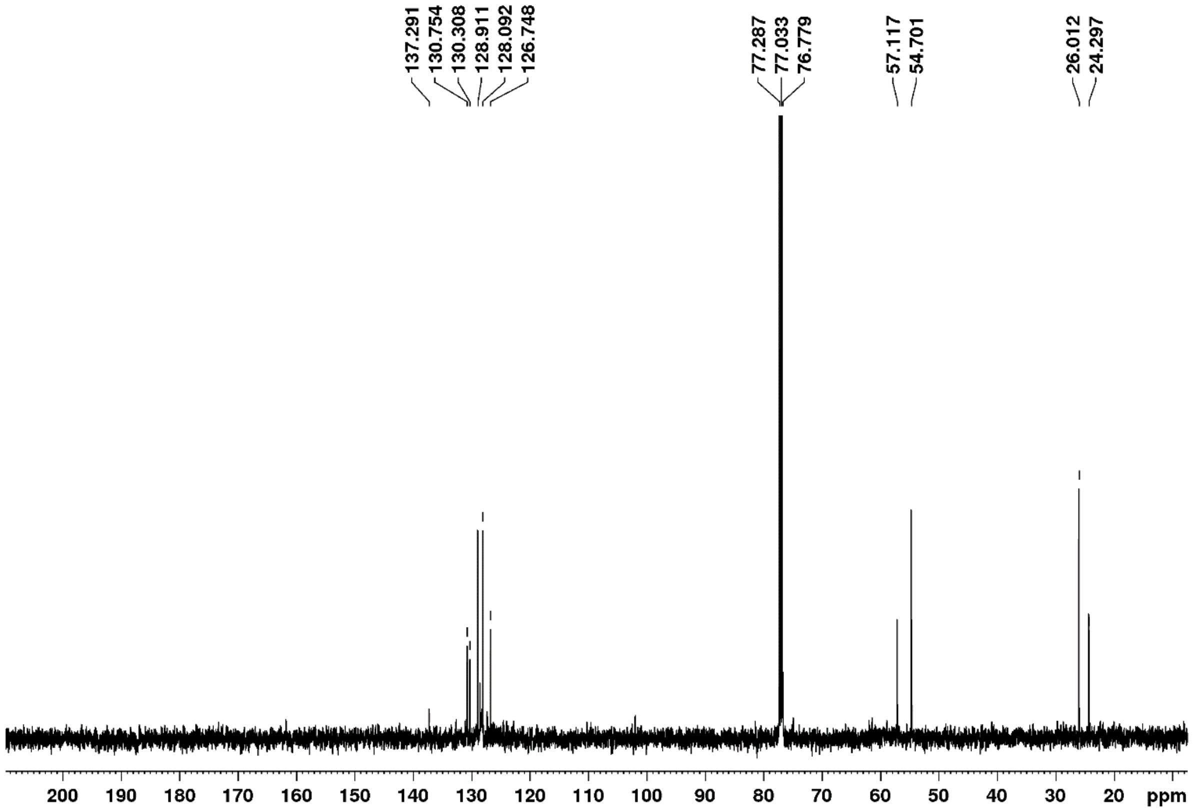

^{1}H-NMR spectrum of (*Z*)-1-(3-phenylallyl)piperidine (2f)

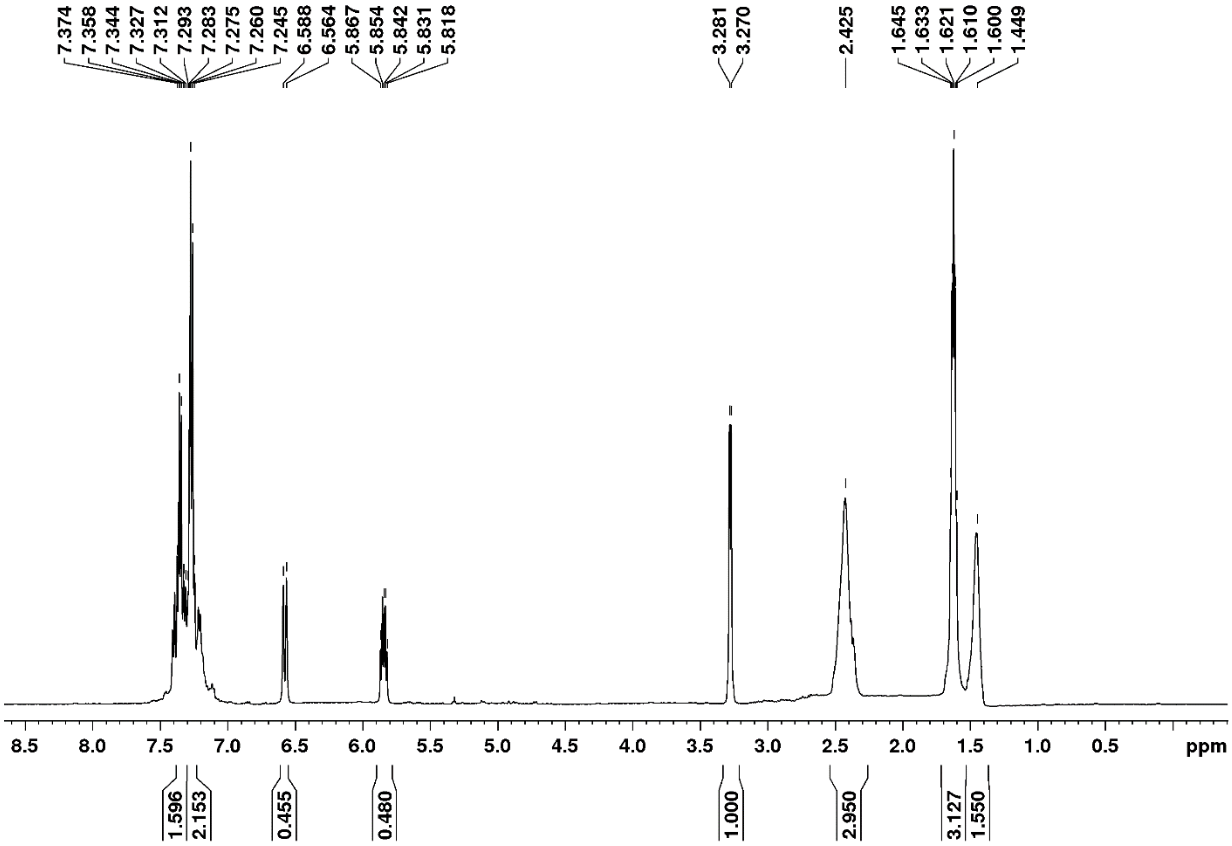

^{13}C-NMR spectrum of (*Z*)-4-(3-phenylallyl-2,3-d_2)morpholine (3e)

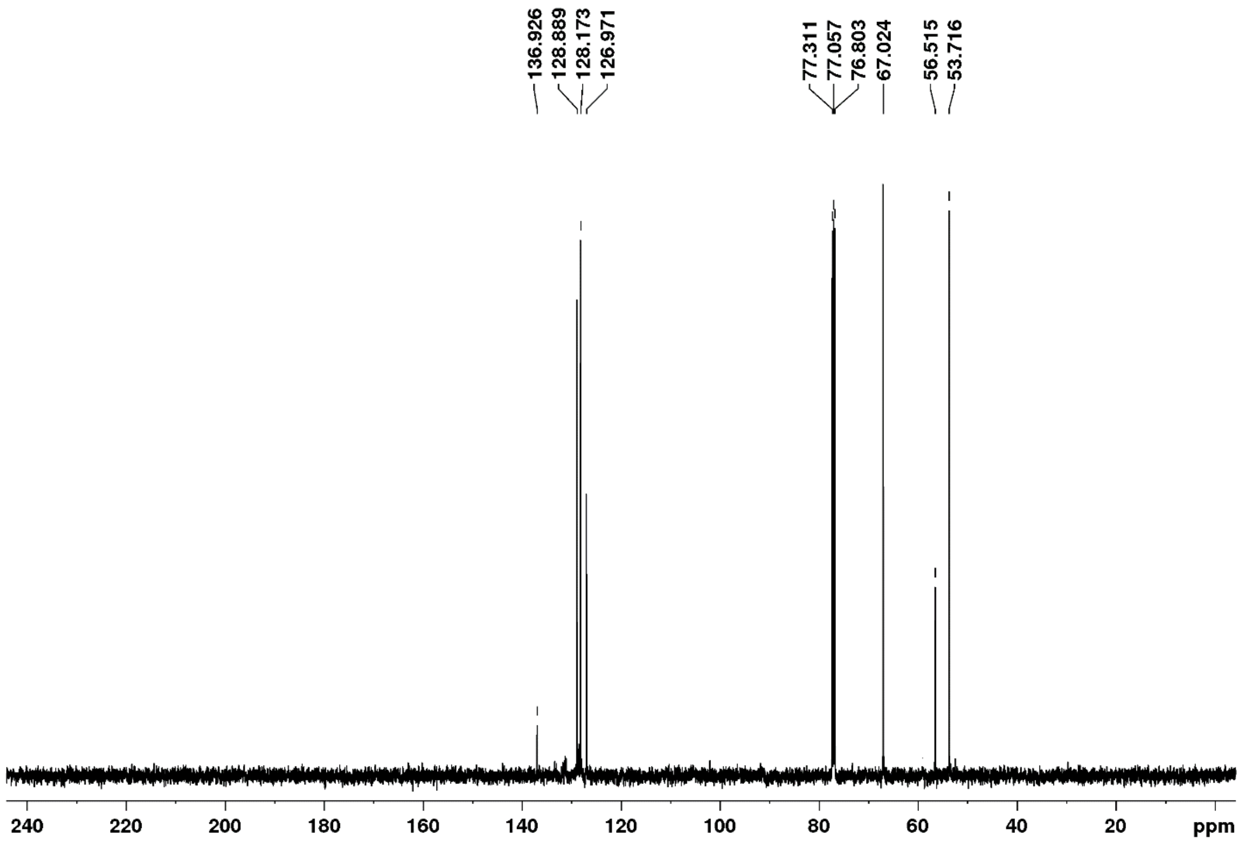

^{1}H-NMR spectrum of (*Z*)-4-(3-phenylallyl-2,3-d_2)morpholine (3e)

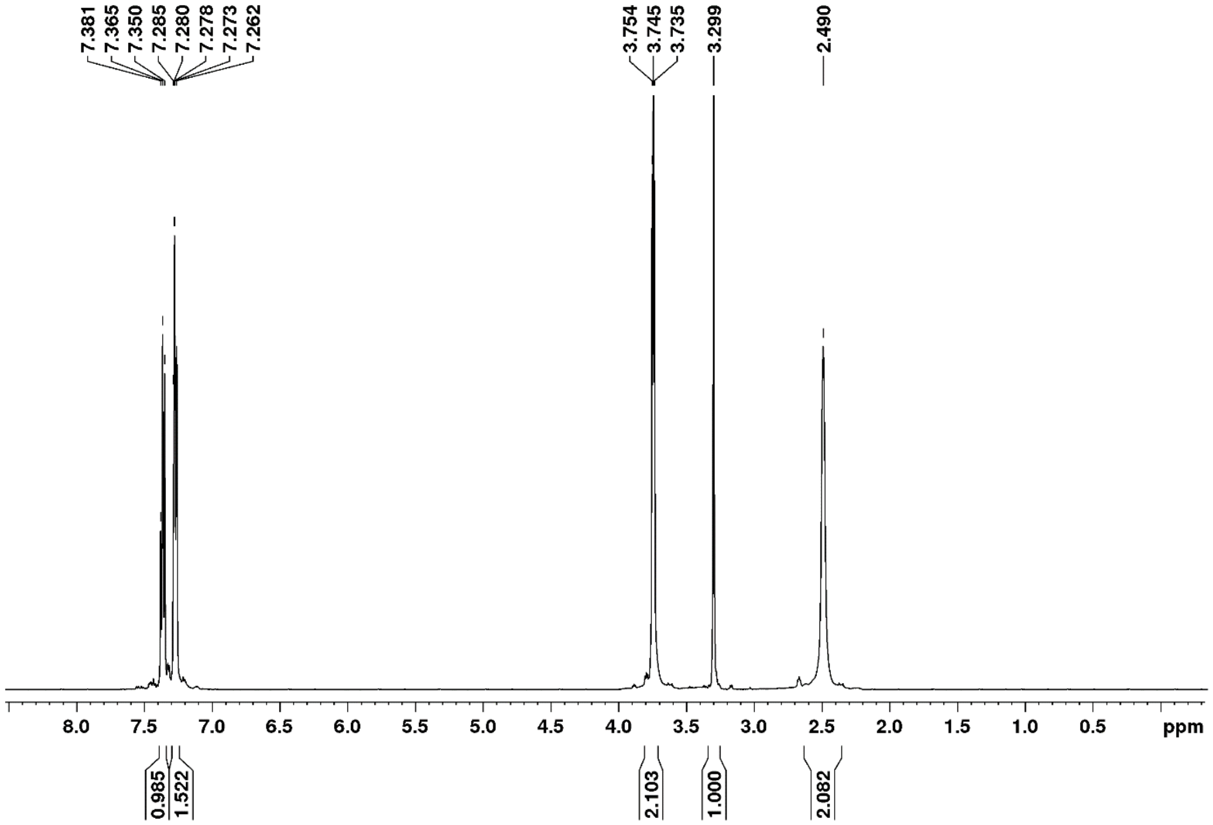

^{13}C-NMR spectrum of (*Z*)-4-(hept-2-en-1-yl-2,3-d_2)morpholine (3c)

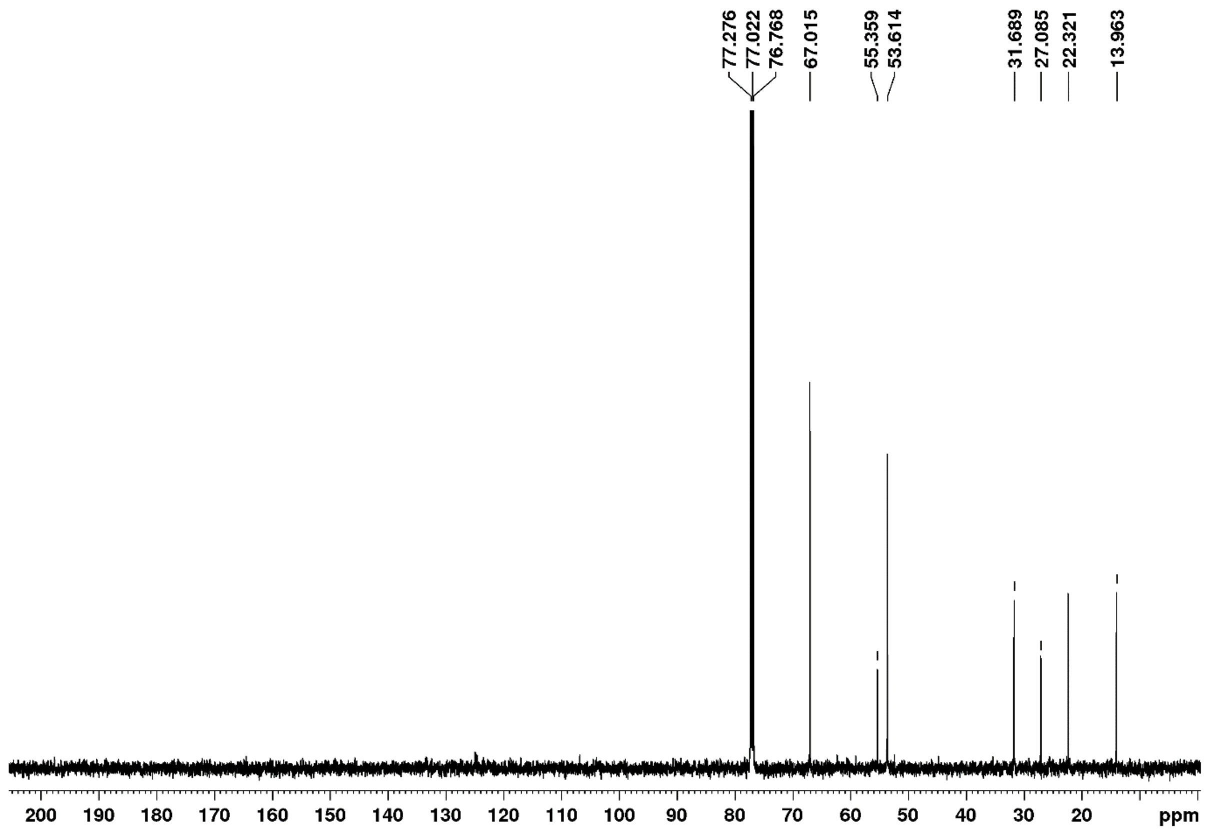

^{1}H-NMR spectrum of (*Z*)-4-(hept-2-en-1-yl-2,3-d_2)morpholine (3c)

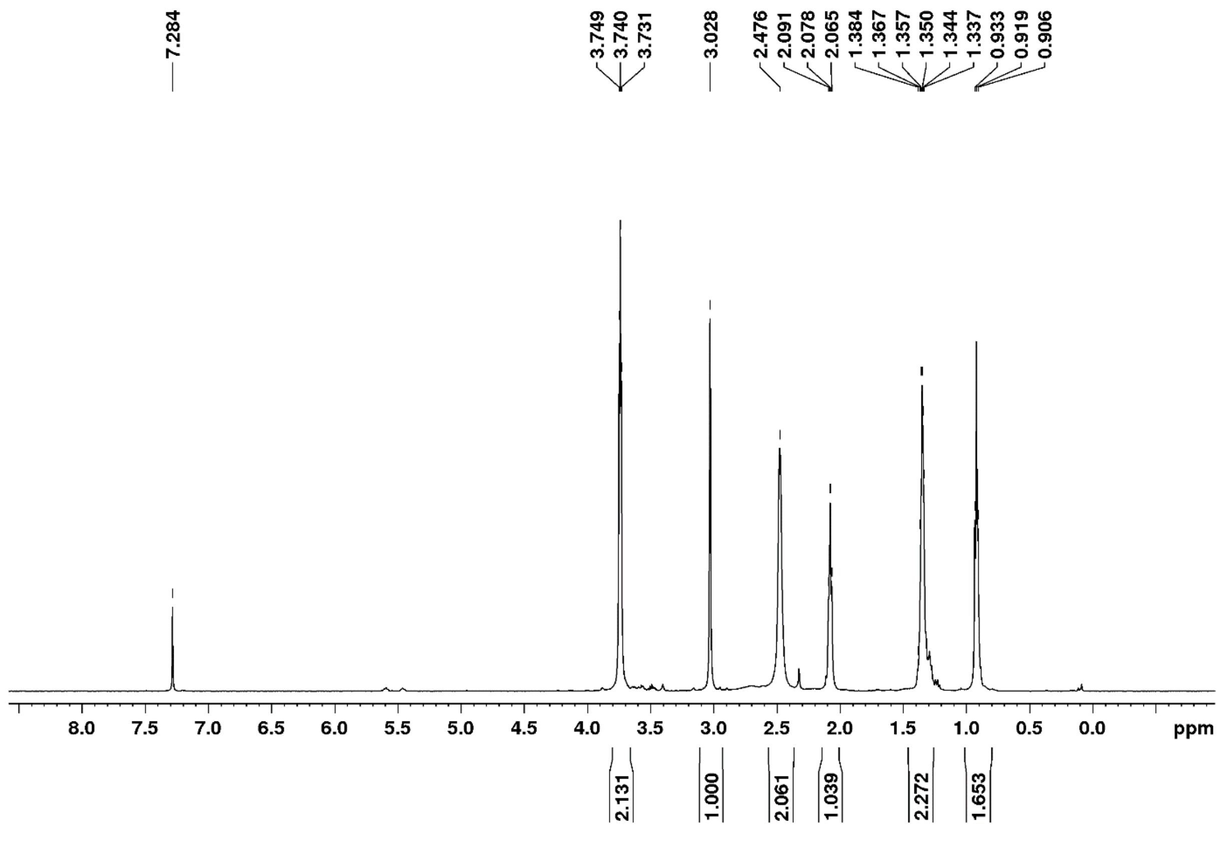

^{13}C-NMR spectrum of (*Z*)-4-phenylbut-3-en-1-ol (5e)

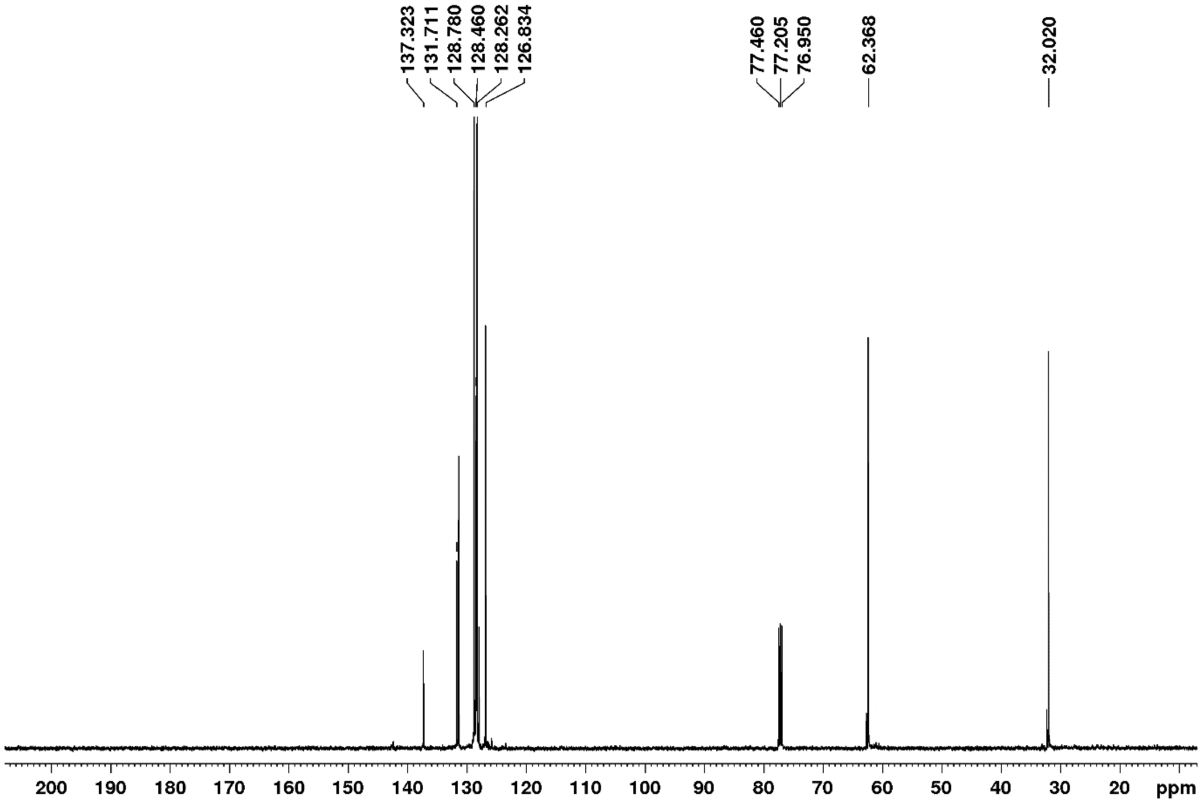

^{1}H-NMR spectrum of (*Z*)-4-phenylbut-3-en-1-ol (5e)

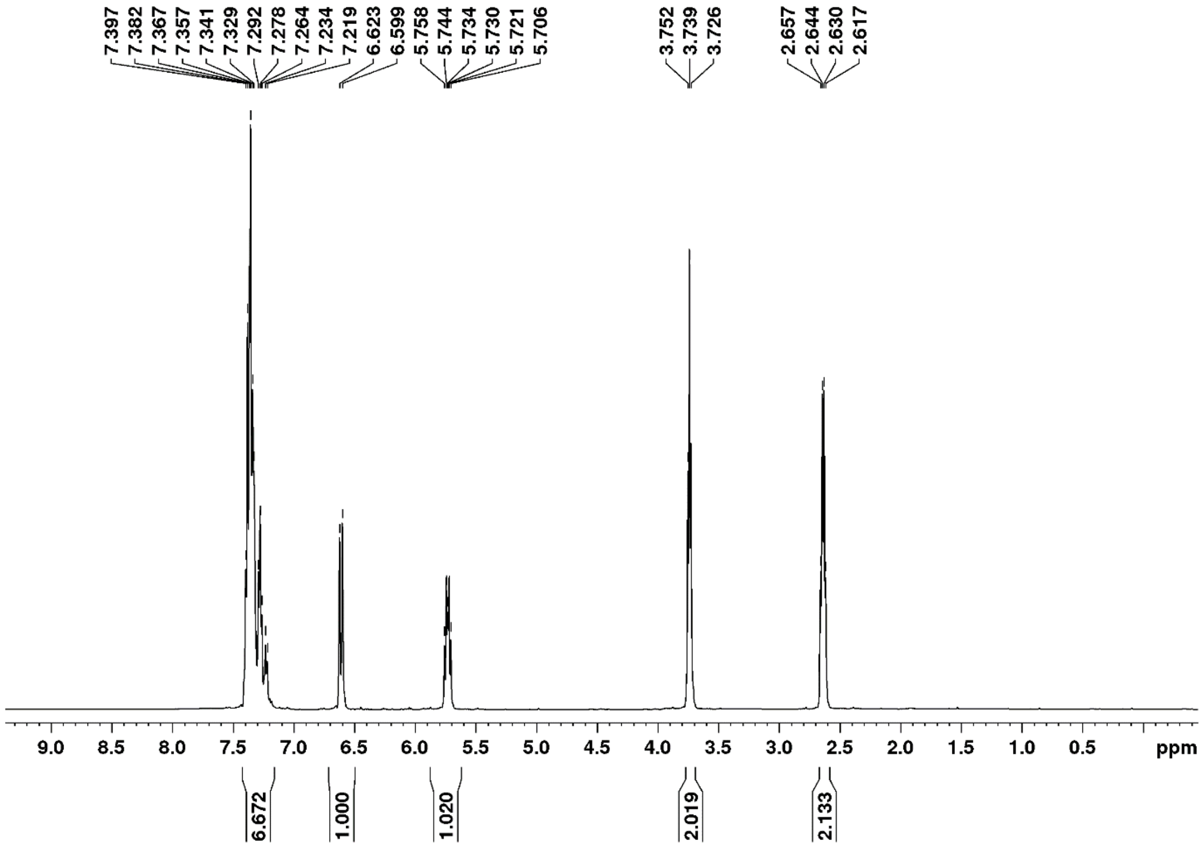

^{13}C-NMR spectrum of (*Z*)-dec-3-en-1-ol (5a)

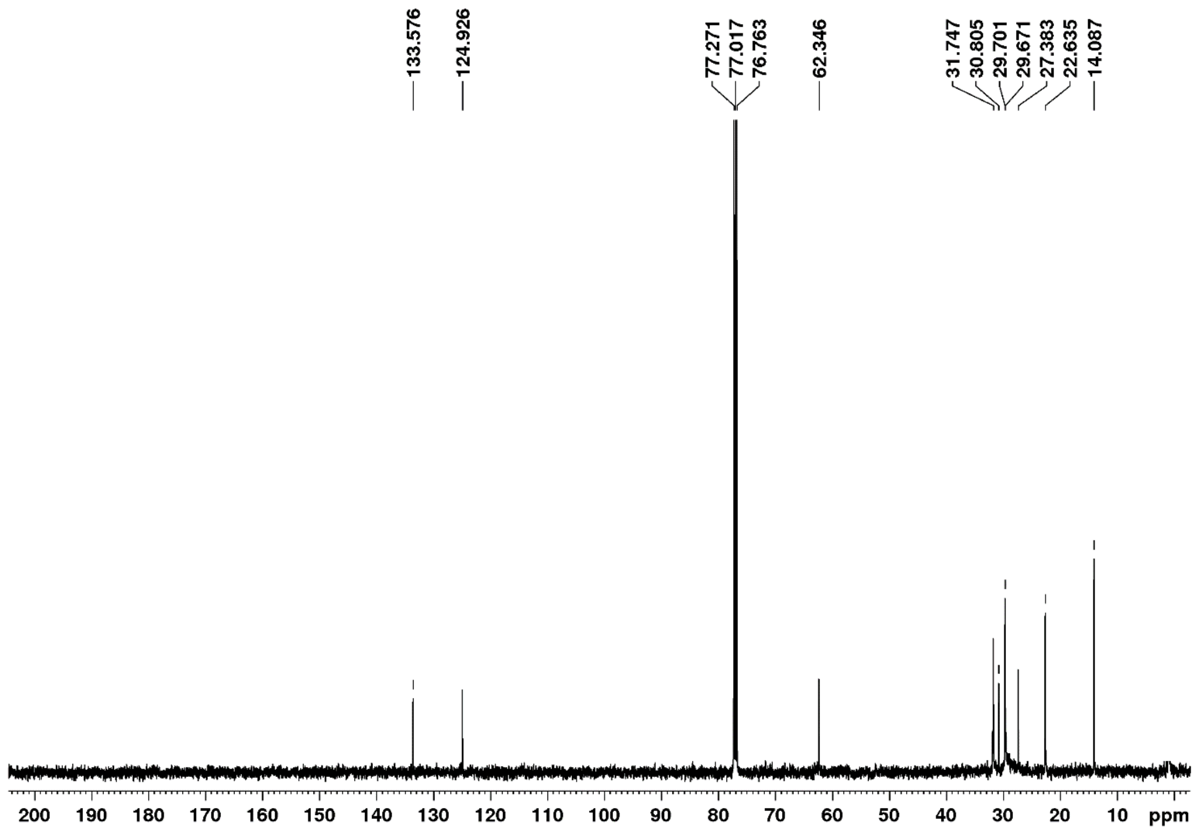

^{1}H-NMR spectrum of (*Z*)-dec-3-en-1-ol (5a)

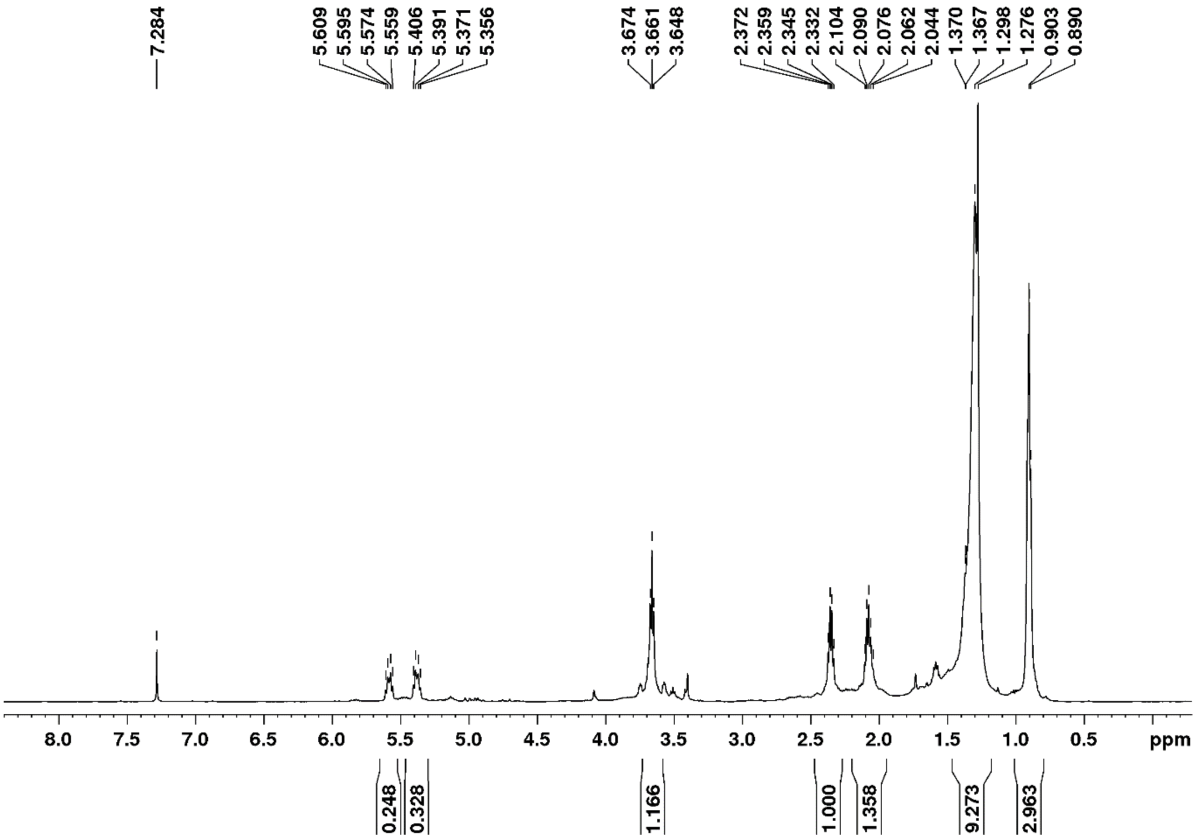

^{13}C-NMR spectrum of (*E*)-4-(2-chloro-3-(methylthio)hept-2-en-1-yl)morpholine (6c)

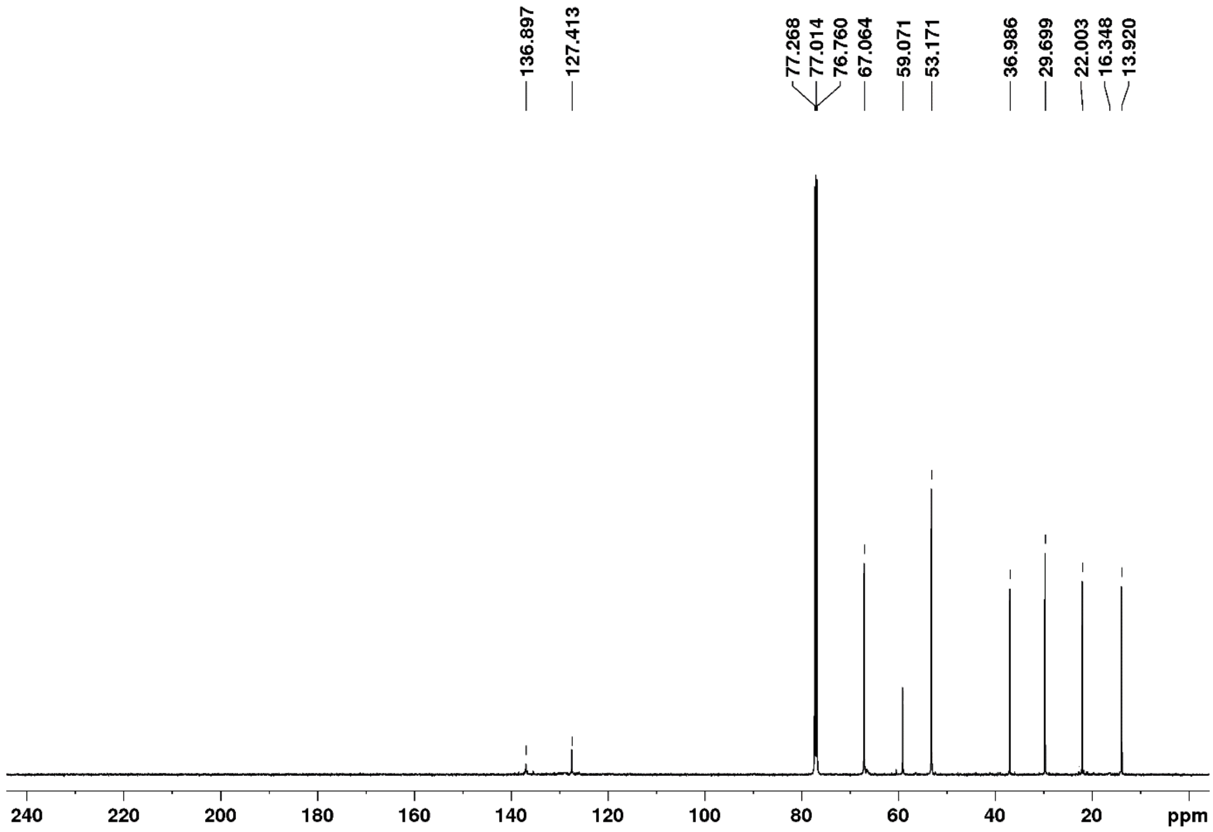

^{1}H-NMR spectrum of (*E*)-4-(2-chloro-3-(methylthio)hept-2-en-1-yl)morpholine (6c)

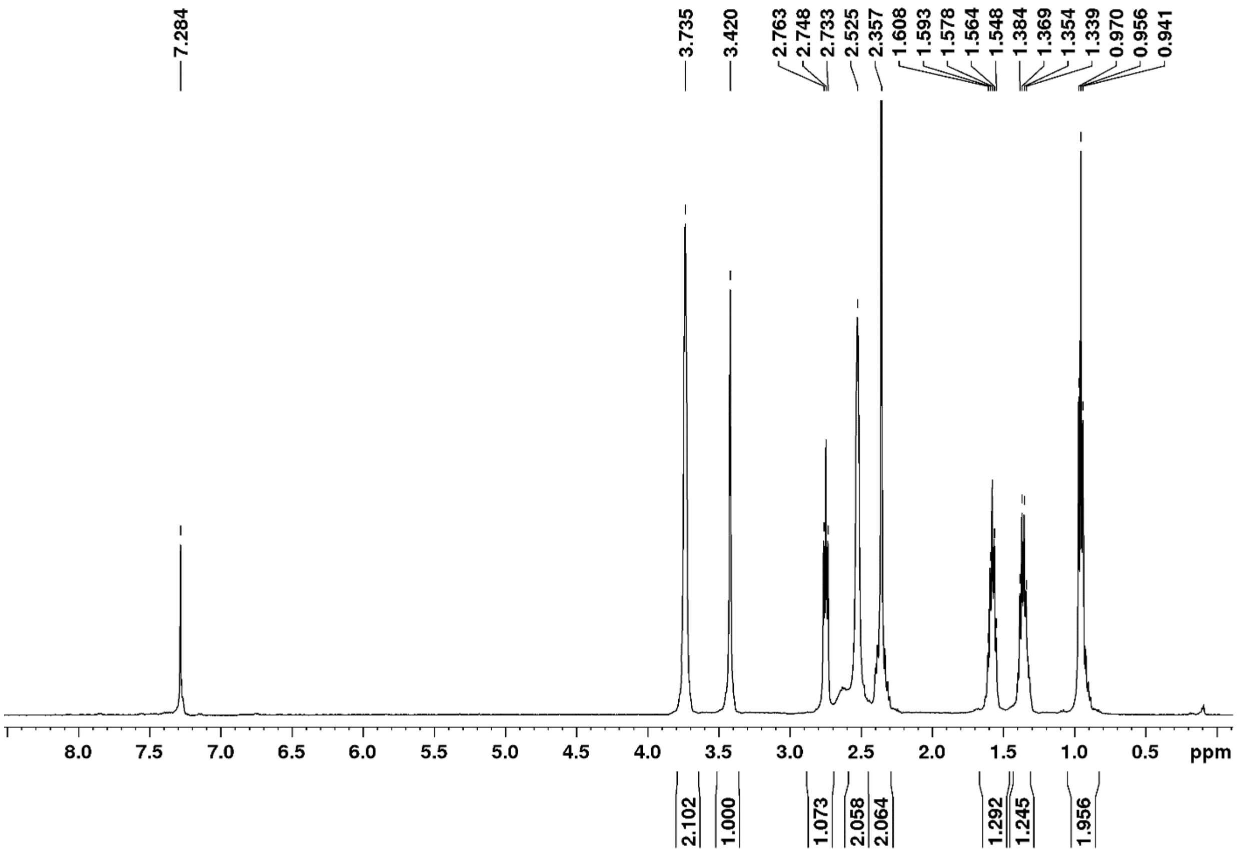

^{13}C-NMR spectrum of (*E*)-1-(2-chloro-3-(methylthio)hept-2-en-1-yl)piperidine (6b)

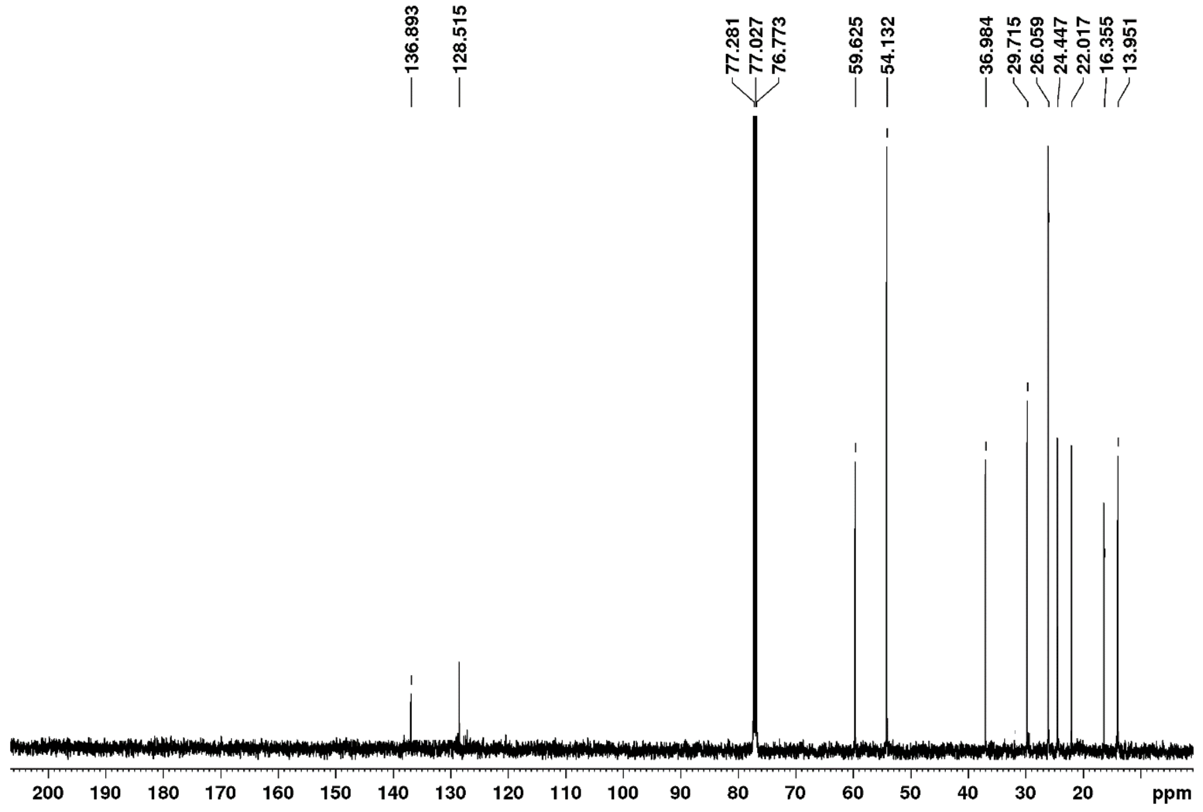

^{1}H-NMR spectrum of (*E*)-1-(2-chloro-3-(methylthio)hept-2-en-1-yl)piperidine (6b)

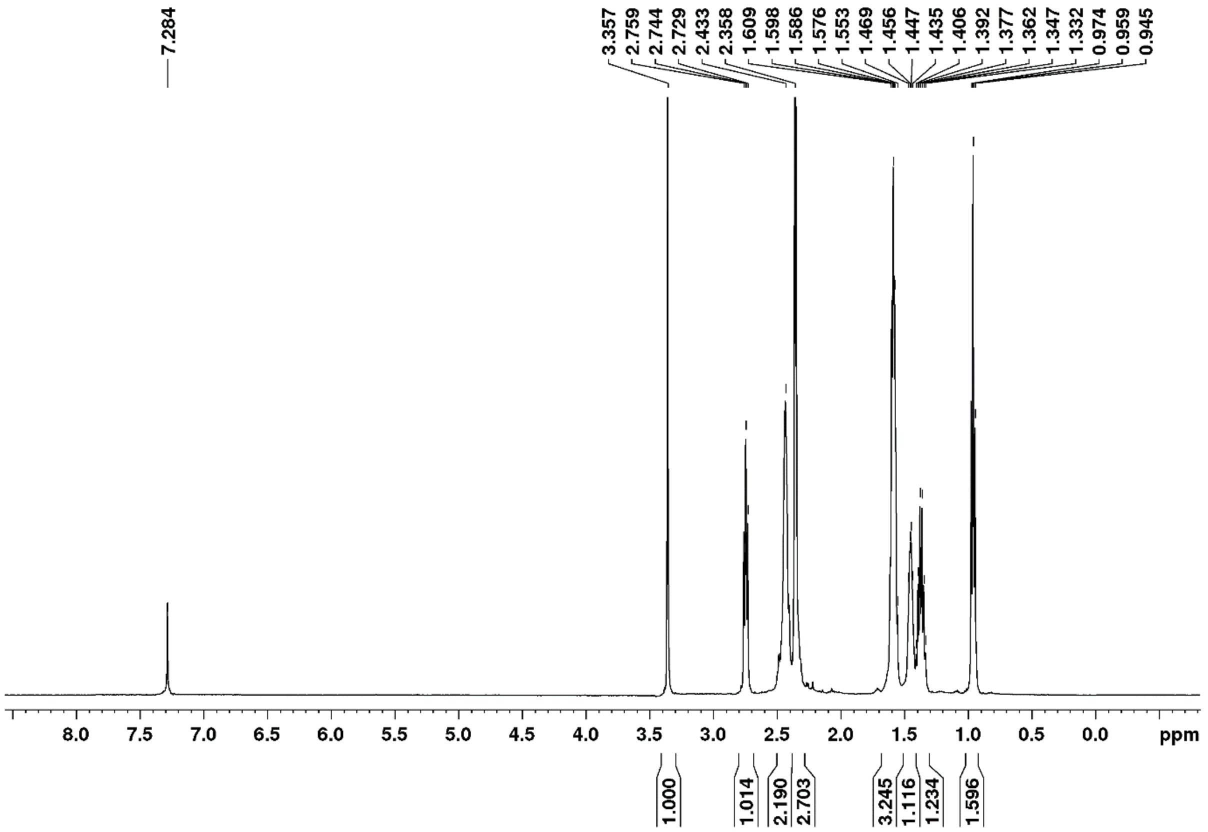

^{13}C-NMR spectrum of (*E*)-4-(2-chloro-3-(methylthio)non-2-en-1-yl)morpholine (6a)

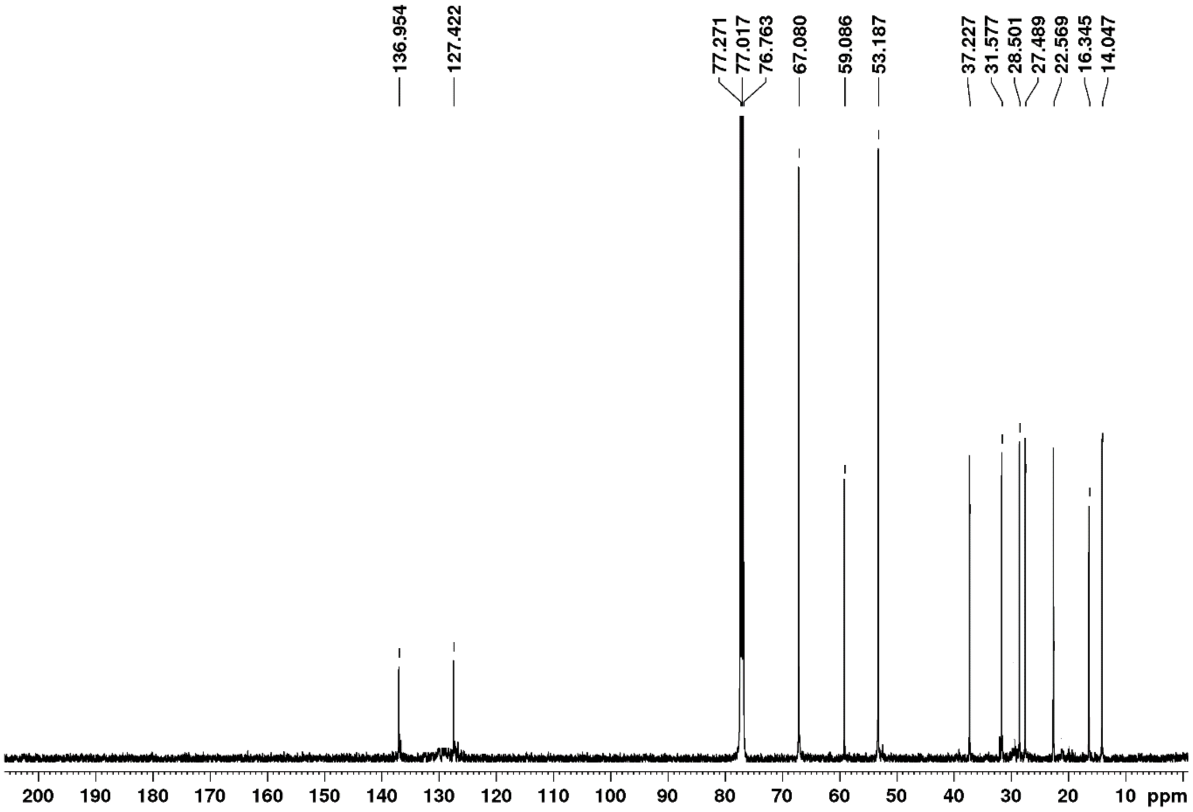

^{1}H-NMR spectrum of (*E*)-4-(2-chloro-3-(methylthio)non-2-en-1-yl)morpholine (6a)

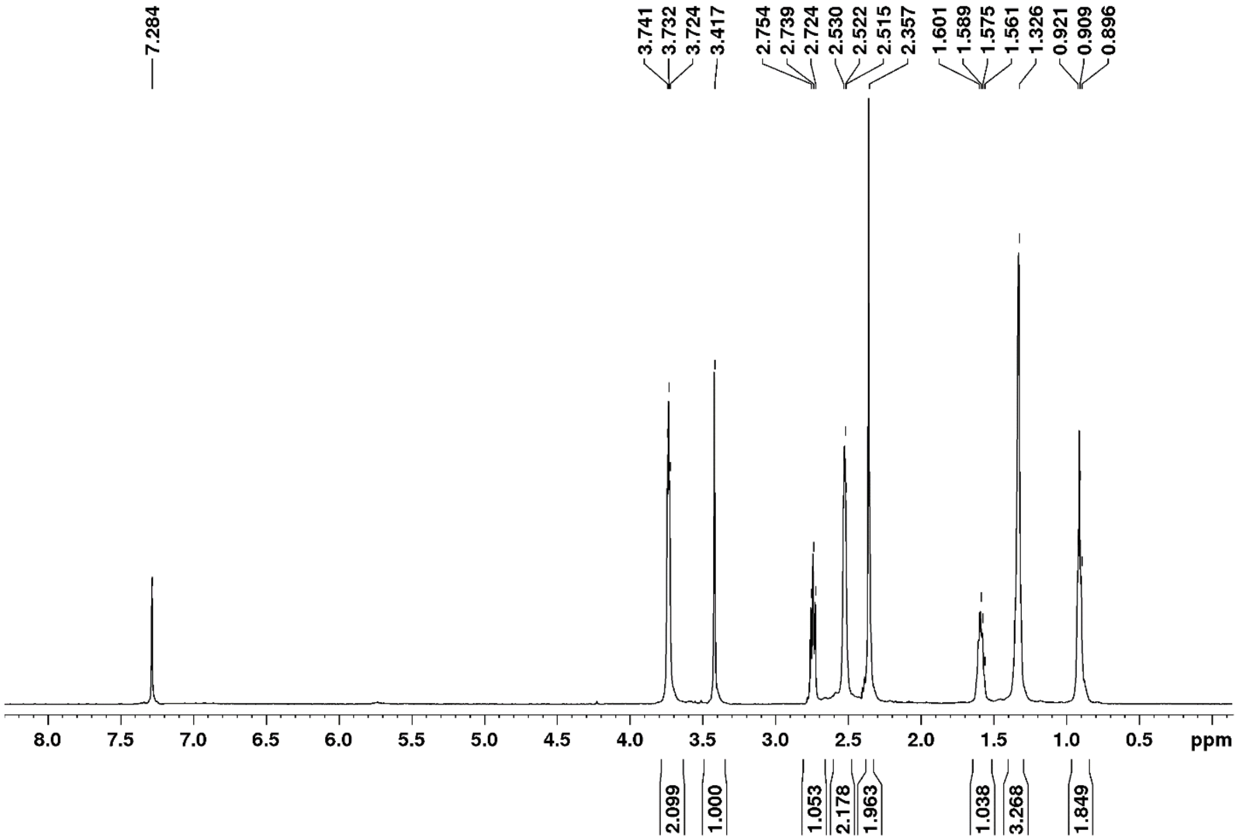

^{13}C-NMR spectrum of (*Z*)-*N*,*N*-dibenzylnon-2-en-1-amine (**2g**)

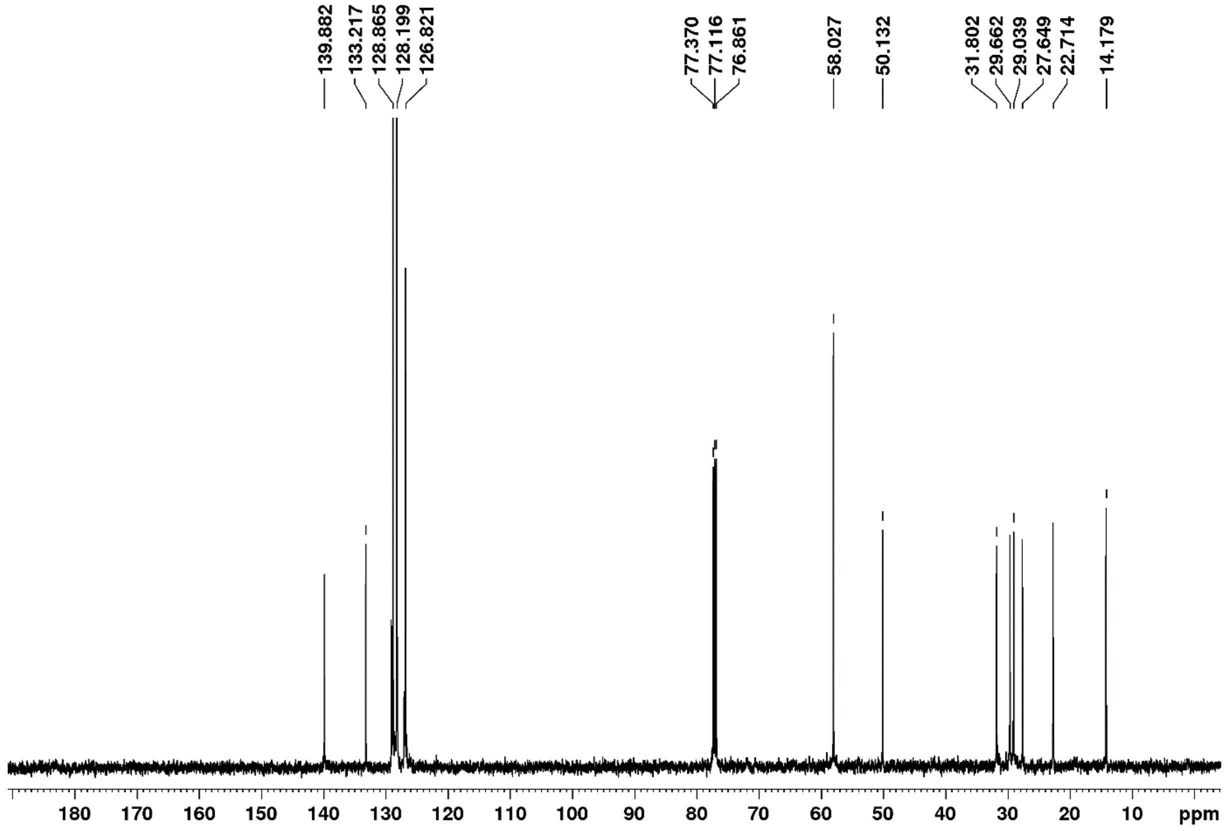

^{1}H-NMR spectrum of (*Z*)-*N*,*N*-dibenzylnon-2-en-1-amine (**2g**)

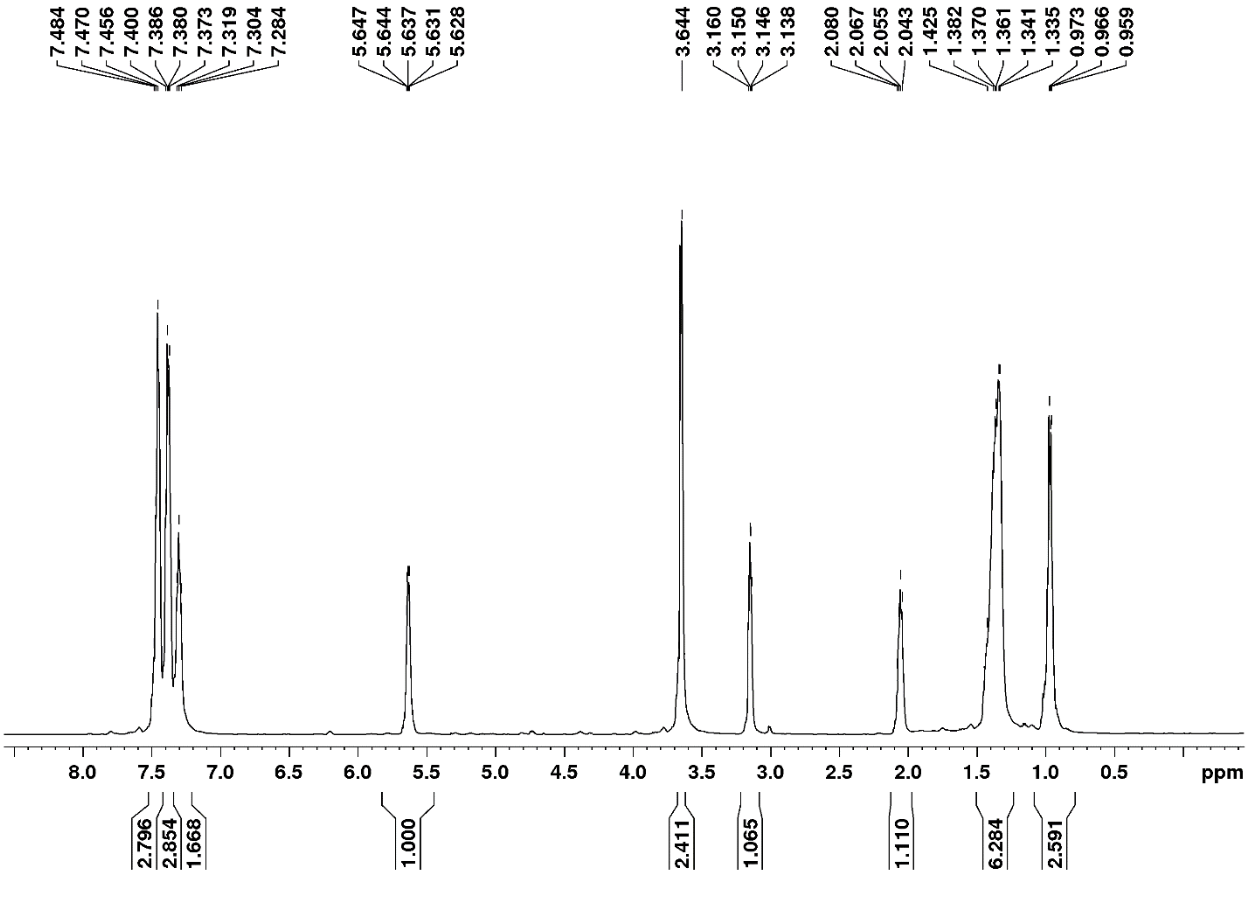

^{13}C-NMR spectrum of (*Z*)-*N*-(3-phenylallyl)butan-1-amine (**2h**)

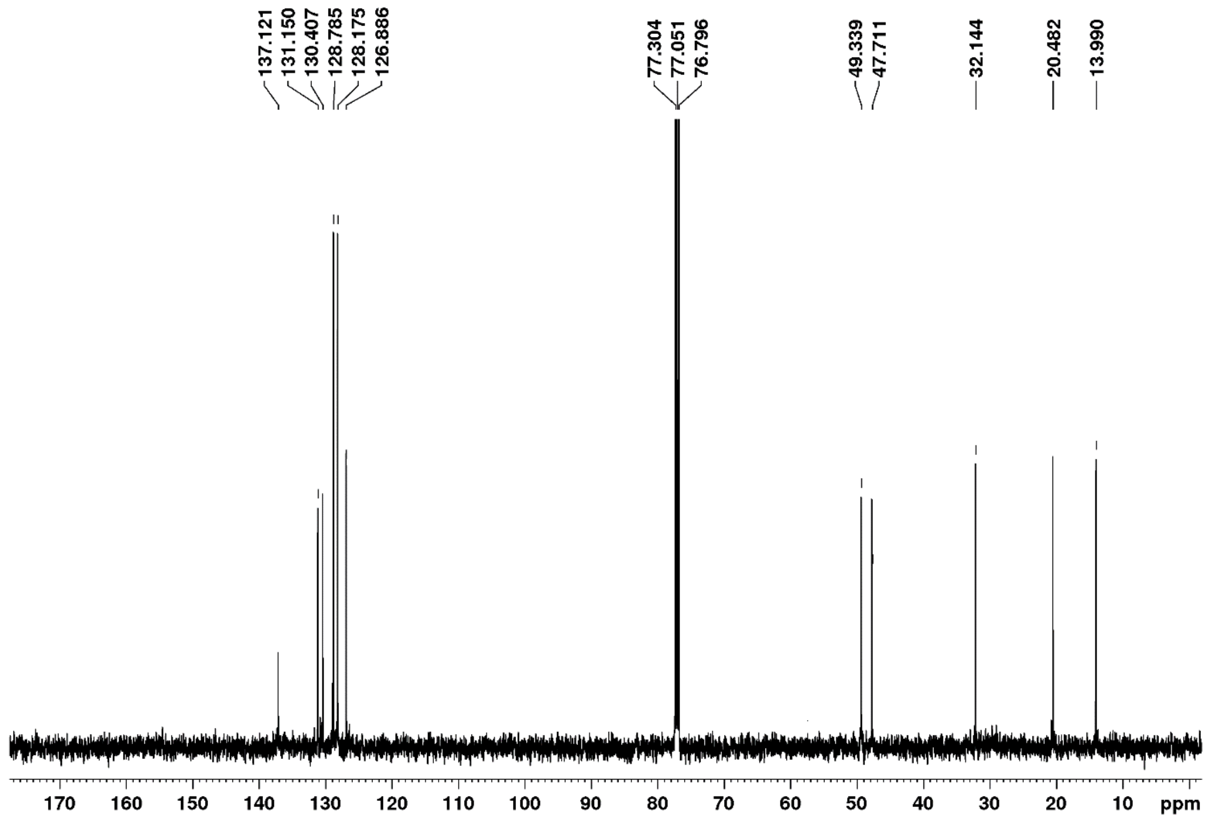

^{1}H-NMR spectrum of (*Z*)-*N*-(3-phenylallyl)butan-1-amine (**2h**)

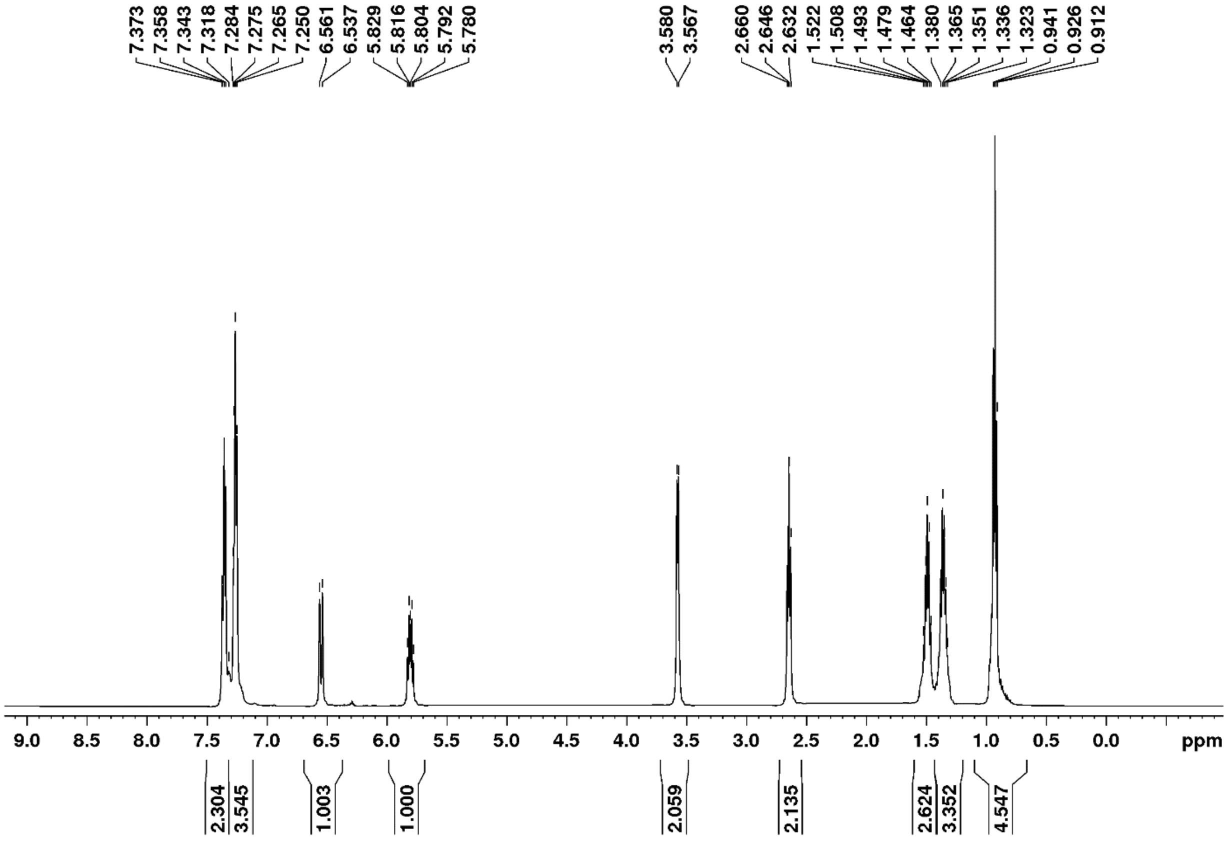

^{13}C-NMR spectrum of (*Z*)-oct-3-en-1-ol (**5c**)

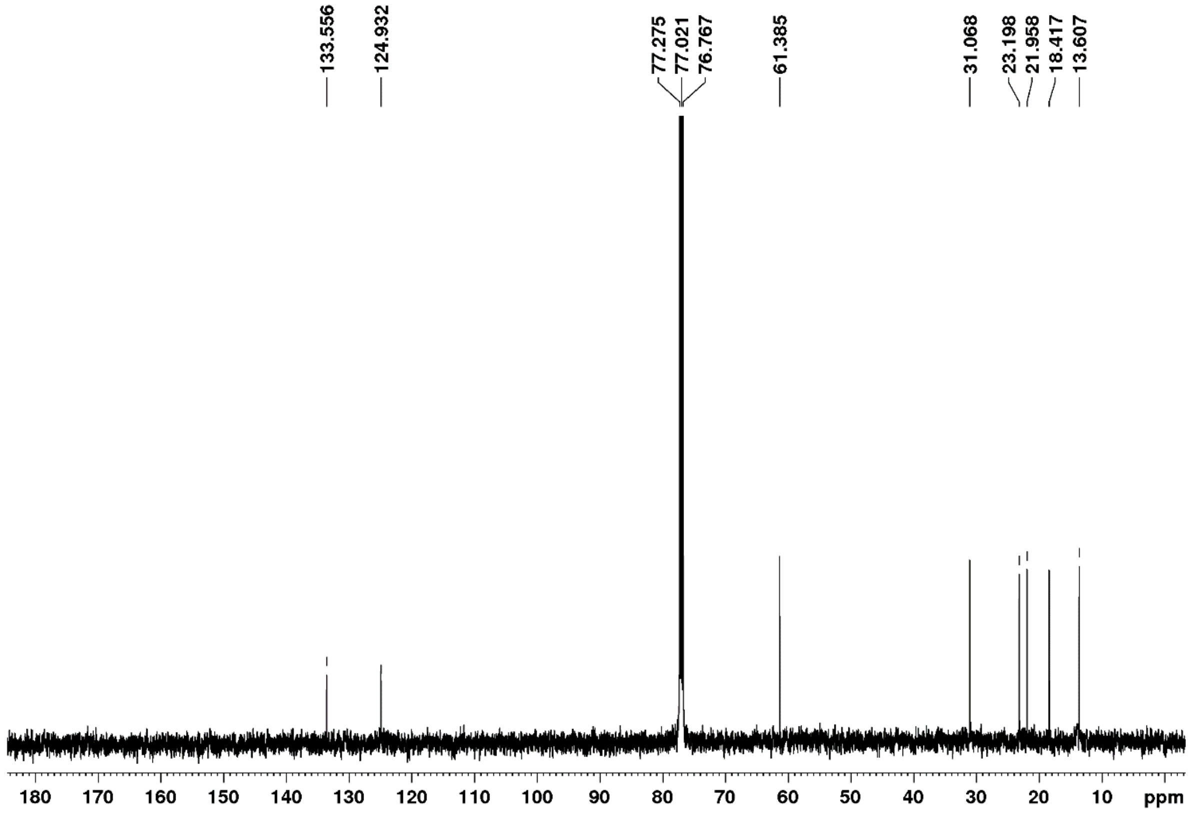

^{1}H-NMR spectrum of (*Z*)-oct-3-en-1-ol (**5c**)

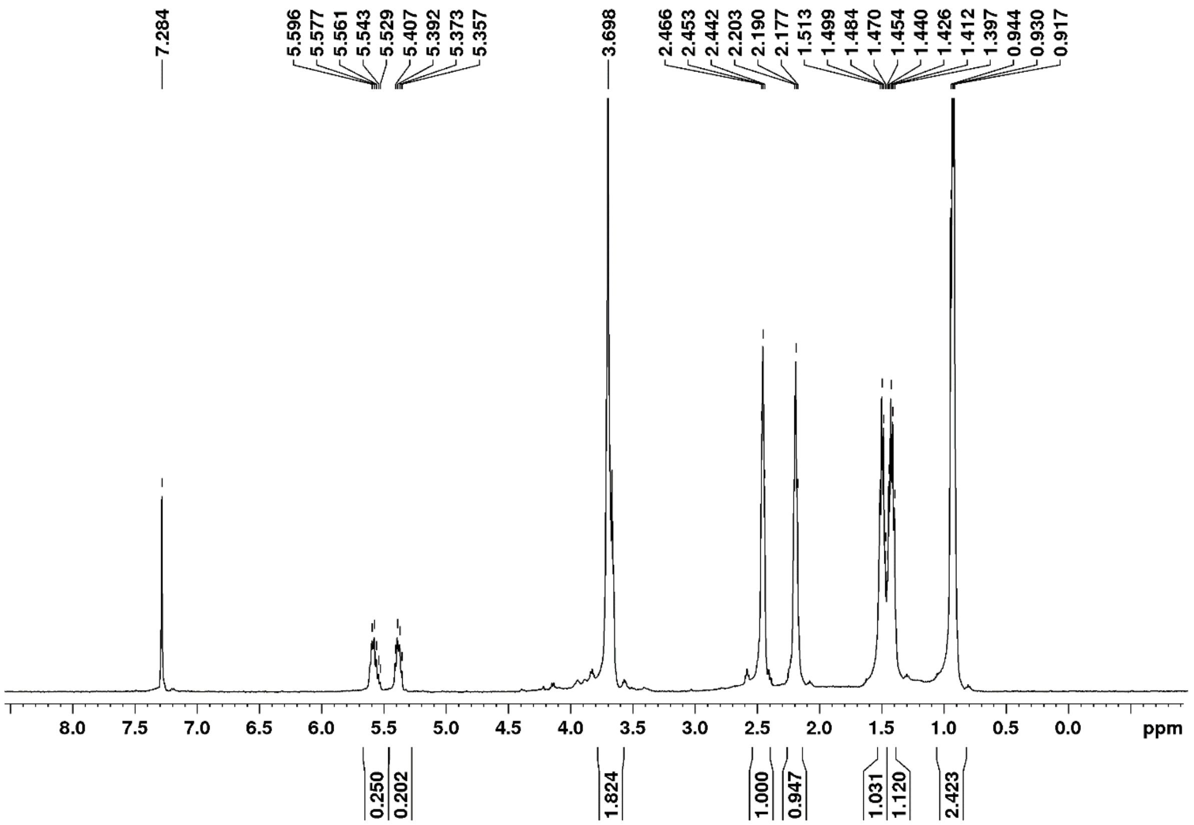

Supplement: Supplementary file 1 [file molecules-26-03722-s001.zip › molecules-1245488-supplementary.pdf]
